# Supplementary material for: Mitochondrial DNA disease discovery through evaluation of genotype and phenotype data: The Solve-RD experience
Source: Am J Hum Genet. 2025 Apr 29;112(6):1376–87. doi: 10.1016/j.ajhg.2025.04.003 (PMC12256788; doi:10.1016/j.ajhg.2025.04.003)
Supplement: Document S1. Figures S1–S6, Table S1, supplemental note, supplemental methods, and supplemental results [file mmc1.pdf]

The American Journal of Human Genetics, Volume *112*

Supplemental information

Mitochondrial DNA disease discovery through evaluation of genotype and phenotype data: The Solve-RD experience

Thiloka Ratnaike, Ida Paramonov, Catarina Olimpio, Alexander Hoischen, Sergi Beltran, Leslie Matalonga, Solve-RD Consortium, and Rita Horváth

Supplemental Information

Supplemental Note: Case Reports

Case summaries for individuals with likely causative variants supported by phenotype similarity score

The following data is summarized in **Table S2. P22** carries *NC_012920.1:m.3302A>G*, and presented with epileptic encephalopathy, epileptic spasm, microcephaly, and global developmental delay. The variant was found at 86% heteroplasmy in blood, with a medium phenotype similarity score (0.41), and a recent publication presented a similar individual with myoclonic epilepsy with ragged red fibers (MERRF, OMIM:545000),[8] therefore the variant has been fed back to the recruiting clinical team.

P23, carrying *NC_012920.1:m.8344A>G*, presented with mitochondrial myopathy, progressive muscle weakness, ptosis, and myopathic electromyography. There were also additional terms including hemifacial atrophy and arrhythmia which are not classically seen with MERRF (OMIM:545000), the commonly associated condition with this variant.[8] The blood heteroplasmy was 71%, with a high phenotype similarity (0.53), so the clinical team were made aware of this likely diagnosis.

P24 with *NC_012920.1:m.8993T>G* had an infantile presentation of epilepsy, cerebellar atrophy, increased serum lactate and arachnoid cyst. There was a high blood heteroplasmy of 94%, with medium phenotype similarity (0.44) likely due to the additional phenotype term of ‘arachnoid cyst’, which is probably not related to mitochondrial disease. Given the likelihood of maternally inherited Leigh syndrome[9] in this case (OMIM:500017), it was urgently fed back to the clinical team.

Single system conditions: **P25-27** were found to have near-homoplasmic variants known to cause LHON[10] (OMIM:535000), with high phenotype similarity in each case (1.0): *NC_012920.1:m.11778G>A, NC_012920.1:m.14484T>C, NC_012920.1:m.3460G>A.* **P28-37** were found to have HPO terms related to sensorineural hearing impairment leading to high phenotype similarity (0.57-0.58). The variant *NC_012920.1:m.1555A>G* was found at 93%-100% blood heteroplasmy levels. One individual was noted to have a phenotype extending beyond hearing impairment with microcephaly and global developmental delay, therefore the *NC_012920.1:m.1555A>G* is only partially explaining this person's phenotype. The variants were fed back via the DITF, and the data is available in **Table S2**.

Variants not in MitoPhen

There were 29 individuals with variants not found in MitoPhen (P19-21 and P38-63 in **Table S2**). 6/29 (21%) had high PSS (>0.5) although the variants were classified as VUS, 2/6 (33%) were diagnostic (P19 and P20, **Table S2**). The variants identified in individuals 19 and 20 in our pipeline, classified as VUS by our team, had also been tagged as causative in the GPAP in parallel. Individual 19 was diagnosed with the *NC_012920.1:m.5698G>A* variant with a blood heteroplasmy of 49%, and a high PSS (0.64) due to a phenotype in keeping with a mitochondrial myopathy including terms: ragged-red muscle fibers, ptosis, external ophthalmoplegia and proximal muscle weakness. A previous study reported a person with a similar presentation, with the single muscle fiber study confirming segregation of the variant to fibers with mitochondrial dysfunction.[11] Therefore, the individual in our study is the second to be reported with the same diagnosis.[12] P20 was diagnosed with the *NC_012920.1:m.9032T>C* variant at 84% blood heteroplasmy, high PSS (0.51) with phenotypic terms indicating a multisystem condition: muscular hypotonia, motor delay,

constipation, feeding difficulties in infancy, and bruxism. Previous publications have indicated that this variant is associated with neurogenic muscle weakness, ataxia and retinitis pigmentosa[13] or a neurodevelopmental disorder with a broader spectrum,[14,15] at high blood heteroplasmy levels.

P38 carried the *NC_012920.1:m.16023G>A* variant at 7.6% blood heteroplasmy, with a high PSS (0.92) due to a range of phenotypes including hearing impairment, pes cavus, cardiac anomalies, mild global developmental delay, cleft palate, and hypospadias. This variant was classified as a VUS and we have reported this finding back to the referring team to gain further information about segregation of the variant. Previous reports of this variant suggest differing phenotypic spectrums.[16,17] P39, who presented with hypotonia and seizures, was found to have the *NC_012920.1:m.3249G>A* variant, at a blood heteroplasmy of 4.2%. This variant was also classified as a VUS.

Of the 2 remaining individuals with variants associated with high PSS, one variant was classified as likely benign (P40 with *NC_012920.1:m.14696A>G*, PSS was 0.62, heteroplasmy 8%), and P41 had an alternative diagnosis (nuclear gene variant).

There were 19/29 (66%) individuals with a medium PSS. Of note, P21 was diagnosed with the homoplasmic *NC_012920.1:m.9478T>C* variant by the local laboratory associated with a medium PSS (0.33). Although this variant has been reported previously to segregate with Leigh syndrome[18] and APOGEE 2[5] predicts this variant as deleterious, there were no additional lines of evidence to support pathogenicity,[19] so it remains classified a VUS. The *NC_012920.1:m.15990C>T* variant identified in P42 with a PSS of 0.48, was the only variant with a likely pathogenic classification in MITOMAP and by the ClinGen Mitochondrial Disease Nuclear and Mitochondrial Variant Curation Expert Panel classification on ClinVar.[7] However, this variant was at 4% heteroplasmy and the person had an alternative nuclear

genetic diagnosis noted on RD-Connect GPAP. The remaining 4/29 (14%) individuals had low PSS, and the variants were all classified as VUS, so they remain undiagnosed (**Table S2**).

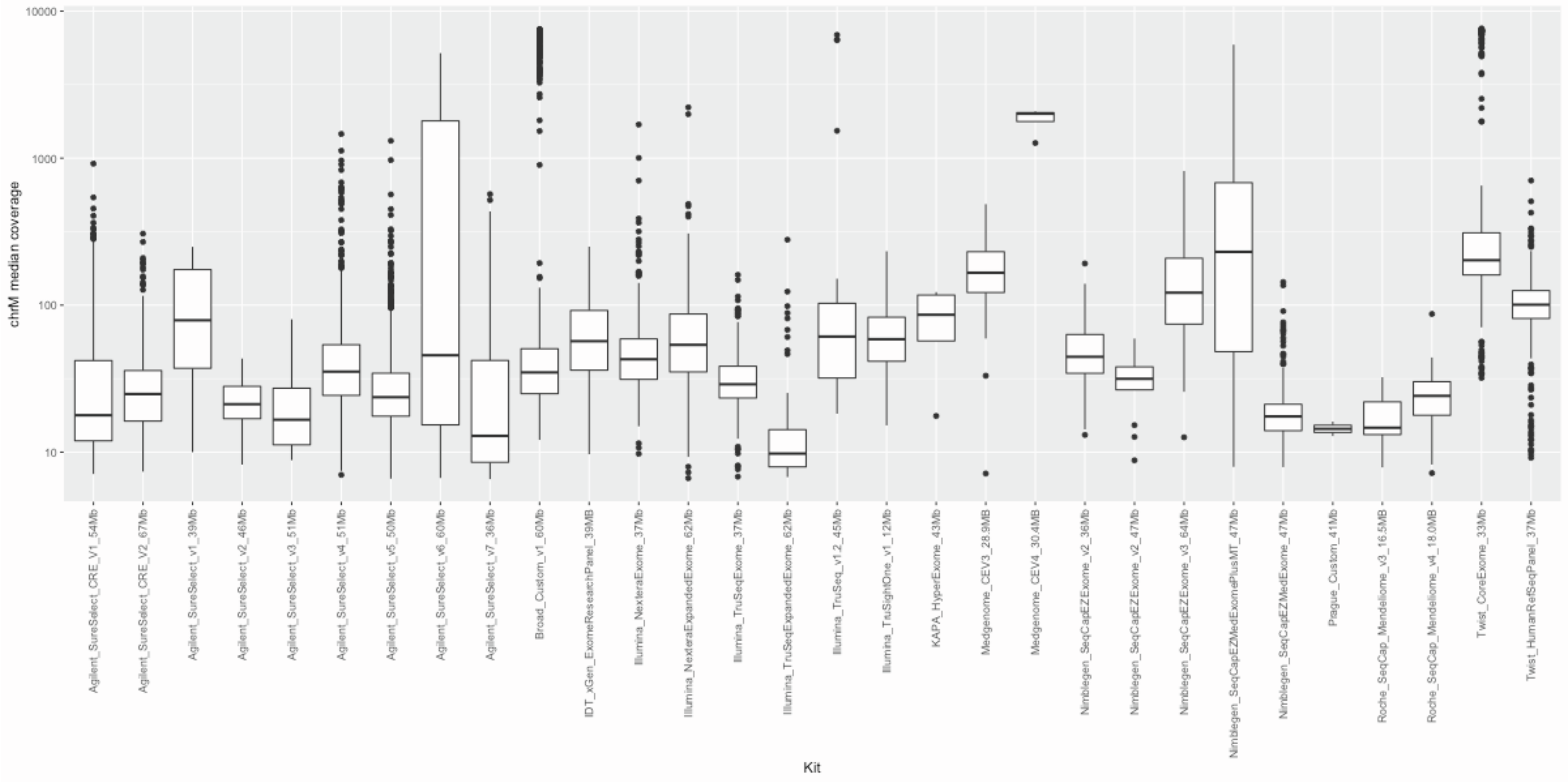

Figure S1: MtDNA coverage across different Exome sequencing (ES) kits after QC filtering.

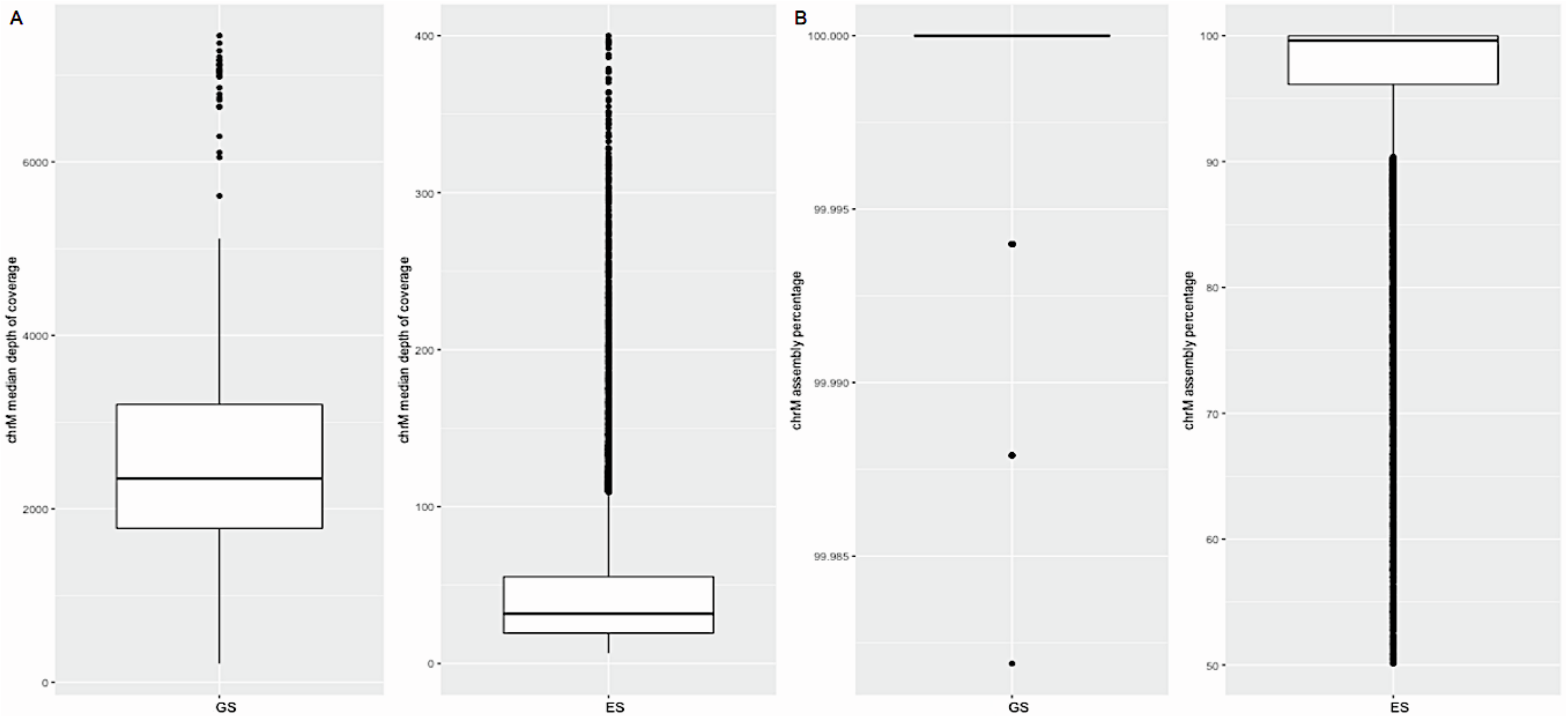

Figure S2: MtDNA coverage and assembly percentage in exome sequencing (ES) and genome sequencing (GS) after QC filtering. A. Median mtDNA coverage in GS (n=523) and ES (n=9,634). B. Percentage of mtDNA assembled in GS (n=523) and ES (n=9,634).

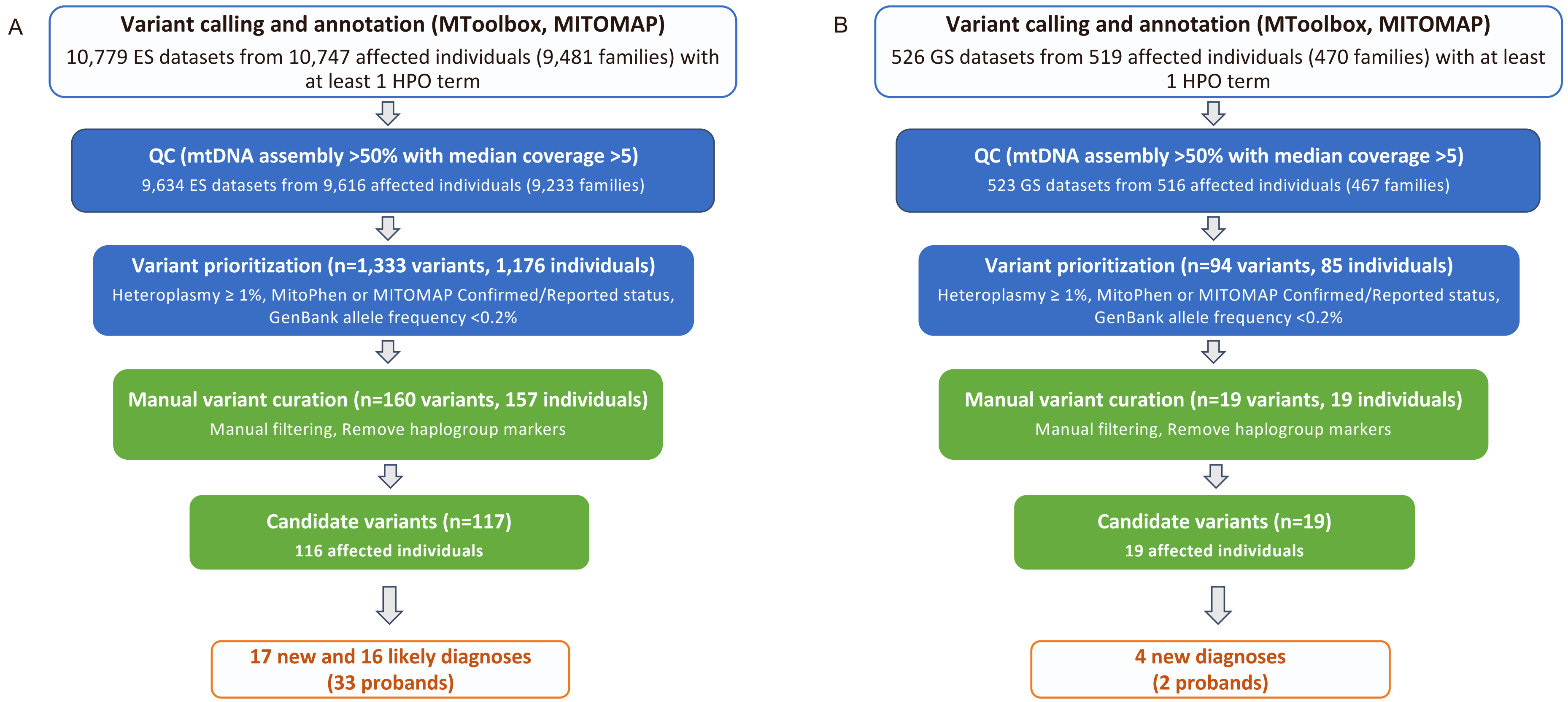

Figure S3: MtDNA variant analysis workflow in exome sequencing (ES) and genome sequencing (GS) datasets. The numbers listed are the number of distinct individual-variant combinations found. Blue and green boxes highlight automated and manual prioritization approaches respectively, with orange highlighting diagnostic/likely causative mtDNA variants. A. Prioritization workflow in 10,779 ES datasets. B. Prioritization workflow in 526 GS datasets.

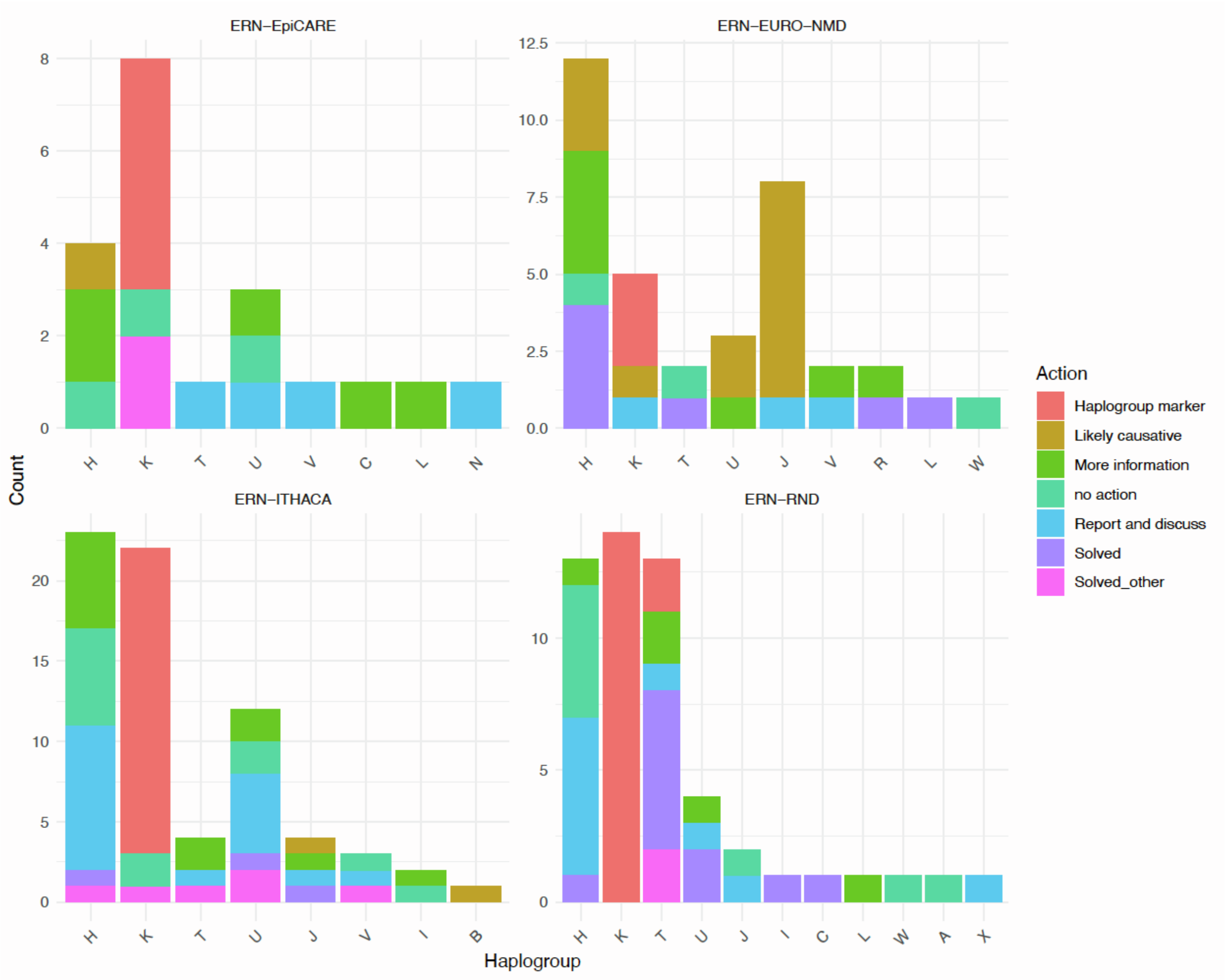

Figure S4: MtDNA haplogroup analysis by ERN. Macro-haplogroup data was used and bars are colored by the final action taken per individual (n=176). The most common haplogroups in the dataset were H, K, J and T. The variant *m.4295A>G* was associated with haplogroup K, and *m.4317A>G* was associated with haplogroup T.

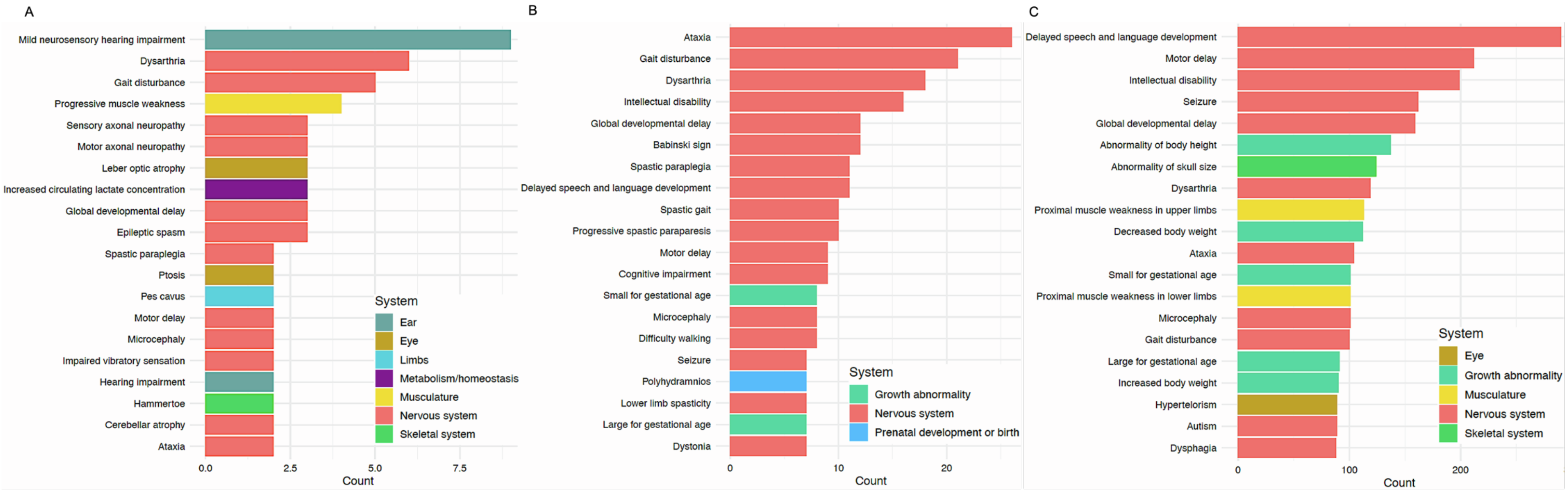

Figure S5: Top 20 frequent HPOs, based on non-redundant terms per diagnosed individual, by genetic diagnosis. A: MtDNA diagnoses (n=37), **B**: Nuclear-mitochondrial gene diagnoses (n=122), **C**: Nuclear-other gene diagnoses (n=1,338).

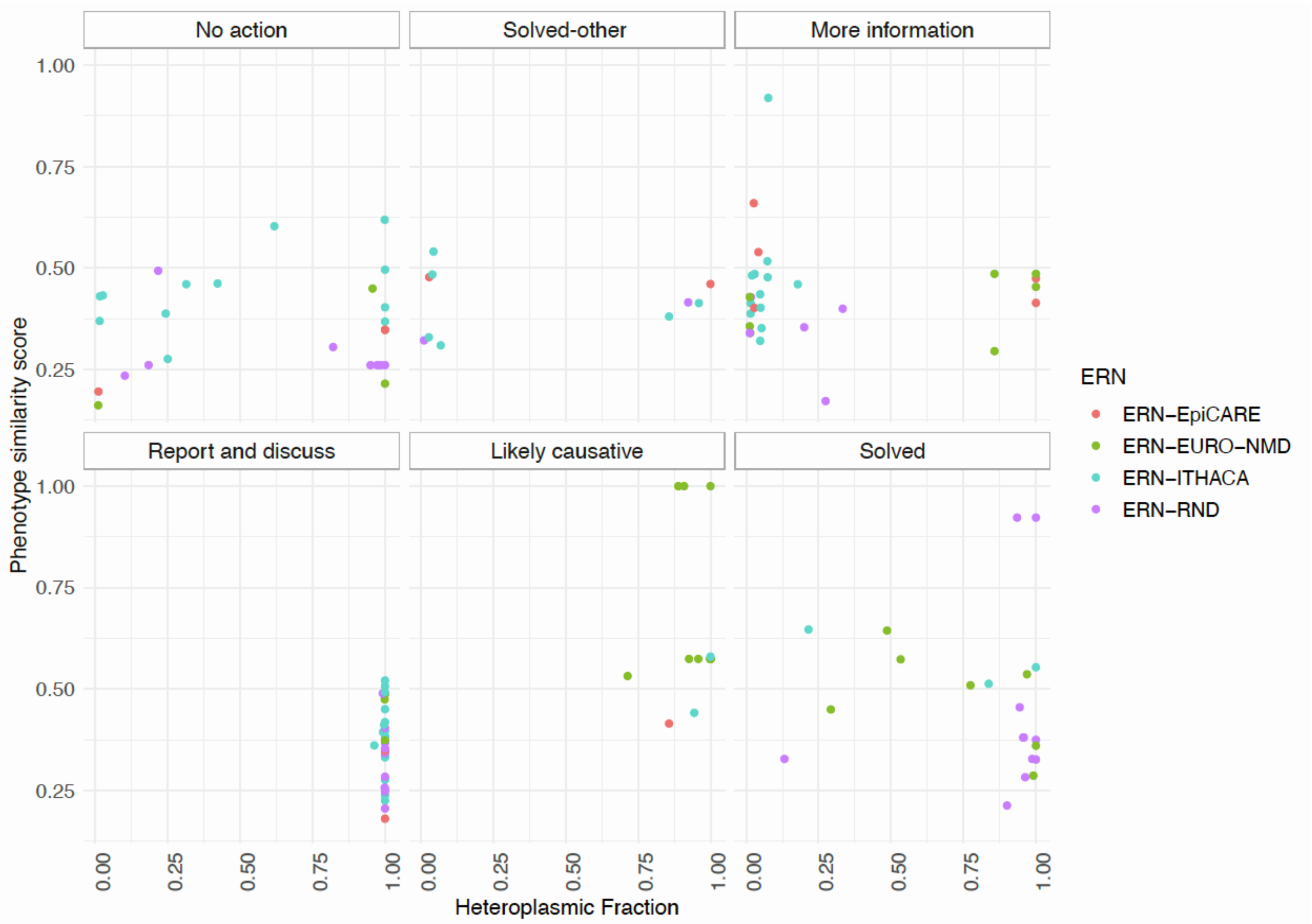

Figure S6: Phenotype similarity score, heteroplasmy level per mtDNA variant, and action taken per sample. The points are colored by ERN that each individual was recruited through.

HGVS nomenclature	Gene, HGNC ID	HF	PSS	ChrM assembled range	Mean chrM coverage range	Number of individuals	ACMG classification (source)
NC_012920.1:m.11778G>A	MT-ND4, HGNC:7459	0.333	1	99.825 - 99.825	42.925 - 42.925	1	Pathogenic (ClinGen)
NC_012920.1:m.11778G>A	MT-ND4, HGNC:7459	0.821	1	99.8914 - 99.8914	24.51 - 24.51	1	Pathogenic (ClinGen)
NC_012920.1:m.11778G>A	MT-ND4, HGNC:7459	0.969	1	99.8733 - 99.8733	40.702 - 40.702	1	Pathogenic (ClinGen)
NC_012920.1:m.11778G>A	MT-ND4, HGNC:7459	1	1	62.46 - 99.994	7.279 - 56.352	7	Pathogenic (ClinGen)
NC_012920.1:m.14484T>C	MT-ND6, HGNC:7462	0.617	1	99.825 - 99.825	42.925 - 42.925	1	Pathogenic (ClinGen)
NC_012920.1:m.14484T>C	MT-ND6, HGNC:7462	1	1	99.6258 - 99.6258	47.353 - 47.353	1	Pathogenic (ClinGen)
NC_012920.1:m.14495A>G	MT-ND6, HGNC:7462	0.935	1	99.9819 - 99.9819	50.729 - 50.729	1	Likely pathogenic (ClinGen)
NC_012920.1:m.14495A>G	MT-ND6, HGNC:7462	0.977	1	99.5896 - 99.5896	33.457 - 33.457	1	Likely pathogenic (ClinGen)
NC_012920.1:m.14674T>C	MT-TE, HGNC:7479	0.98	0.34	99.7707 - 99.7707	68.967 - 68.967	1	Likely pathogenic (ClinGen)
NC_012920.1:m.14674T>C	MT-TE, HGNC:7479	1	0.34	65.3751 - 99.994	13.101 - 165.35	8	Likely pathogenic (ClinGen)
NC_012920.1:m.14674T>C	MT-TE, HGNC:7479	1	0.49	90.1141 - 99.994	17.358 - 155.755	11	Likely pathogenic (ClinGen)
NC_012920.1:m.3460G>A	MT-ND1, HGNC:7455	0.969	1	99.837 - 99.837	56.785 - 56.785	1	Pathogenic (ClinGen)
NC_012920.1:m.3460G>A	MT-ND1, HGNC:7455	1	1	94.1879 - 99.8914	11.75 - 58.473	8	Pathogenic (ClinGen)

Table S1: MtDNA variant benchmarking in 42 individuals with previously confirmed genetic diagnoses (43 confirmed variants – one individual was diagnosed with variants *NC_012920.1:m.11778G>A* and *NC_012920.1:m.14484T>C*). Table is grouped by mtDNA variant, number of individuals are shown per group. HF: heteroplasmic fraction; PSS: phenotype similarity scores; ChrM: Chromosome M or mitochondrial genome.

Supplemental Methods

Mitochondrial DNA manual curation

For mitochondrial DNA (mtDNA) variants not found in MitoPhen, we applied ACMG criteria and tRNA pathogenicity criteria where applicable.[1,2] For pathogenicity assessments, MITOMAP[3], Mitomaster[4] and the Franklin (http://franklin.genoox.com) tool were used to search for publications related to each variant as well as allele frequencies. APOGEE 2[5] and MitoTip[6] were used to determine pathogenicity predictions for variants. Additionally, ClinVar[7], Pubmed and Google searches were conducted per variant of interest to identify any recent publications of individual-level information.

Supplemental Results

MtDNA analysis in the unsolved cohort

From the 11,305 datasets analyzed in the unsolved cohort, 10,157 (90%) have met our quality inclusion criteria with variable coverage across different ES kits (**Figure S1**). In fact, in 523 out of 526 GS initially included in this study, mtDNA was assembled at 100% and median coverage was 2,347 across all (**Figure S2, Figure S3**). From the 10,779 exomes, 9,634 (89%) datasets were considered for further analysis (**Figure S3**). As shown in Figure S2, median mtDNA assembly was 99.6% and median mtDNA coverage was 34 across ES datasets. A small fraction of these exomes (219/9,634, 2.3%) was sequenced using a kit that enables target enrichment of the mitochondrial genome (Nimblegen_SeqCapEZMedExomePlusMT_47Mb). A median coverage across this group was 230, which is higher than in exomes sequenced by other kits (**Figure S1**). Only 6.5% of ES data overall (518/9,634) showed median coverage below 10.

179 variants were identified in 176 affected individuals using the automated prioritization approach initially, prior to exclusion of haplogroup markers. 160 of the prioritized variants were identified using ES and the other 19 were detected from GS (**Figure S3**). The heteroplasmy varied from 1 to 100% both in ES and GS datasets, while median coverage ranged from 8 to 7,369 in ES, and from 1,125 to 6,780 in GS.

Haplogroup analyses

We performed mtDNA haplogroup analysis to identify any haplogroup markers in the dataset at the first step of manual variant curation (**Figure 1**). There were 179 variants considered in 176 individuals: 43 were mtDNA haplogroup markers, with *NC_012920.1:m.4295A>G* being the most frequent, found in 36 individuals associated with haplogroup K, recruited across the four ERNs (**Figure S4**). Therefore, these variants were discarded from further analysis, resulting in 136 rare mtDNA variants in 135 individuals, for manual curation including phenotype evaluation. 5/135 individuals (4%) belonged to Asian (haplogroup C) or African mtDNA lineages (haplogroup L), as denoted in MITOMAP[3], while the rest belonged to mtDNA lineages associated with European ancestry.

Phenotype similarity analysis

When considering all diagnosed individuals within Solve-RD, the frequently documented HPO terms showed that individuals with a mtDNA disease diagnosis tended to have multisystem involvement (**Figure S5A**), compared to those with other nuclear genetic diseases including nuclear-mitochondrial gene diagnoses (**Figure S5B-C**) where phenotypes included more neurodevelopmental presentations such as intellectual disability. Terms related to sensorineural hearing impairment and neuromuscular presentations such as

‘dysarthria’, ‘gait ataxia’ and ‘progressive muscle weakness’, were most frequent in the mtDNA disease cohort. Individuals with a nuclear-mitochondrial genetic diagnosis had a predominance of HPO terms related to nervous system and neurodevelopmental abnormalities, whereas there were more growth abnormality terms seen in the ‘nuclear-other’ group compared with the other two groups. These differences impact the phenotype similarity score (PSS) calculations and enabled the detection of suspected mtDNA diseases within the heterogeneous Solve-RD cohort.

PSS was evaluated alongside mtDNA variant heteroplasmy data in the 136 variants from 135 affected, unsolved individuals that underwent manual curation. 26/136 variants were classified ‘no further action’: 9/26 were variants of uncertain significance (VUS), and reported back to the DITF, but either did not show segregation of variant within the family, or the PSS was low and the phenotype did not match any previous reports; 8/26 were *NC_012920.1:m.1555A>G* with no associated hearing impairment phenotypes and not found in homoplasmy; 9/26 had medium-low PSS associated with variants which were not reported back to the DITF due to conflicting evidence of pathogenicity (*NC_012920.1:m.4317A>G*, *NC_012920.1:m.4295A>G*) or phenotype not matching previous reports (*NC_012920.1:m.15498G>A*).
Of the 37 individuals who had confirmed mtDNA disease diagnoses (n=21) or likely diagnoses (n=16), 34 (92%) had PSS which were >0.3, and all individuals had mtDNA variant heteroplasmy levels >11%. (**Table S2**, **Figure S6**).

MitoPhen update

NC_012920.1:m.4317 will be removed from MitoPhen pathogenic variant list as there is no new supporting evidence, and it is classified as likely benign/benign. The variant classified as likely pathogenic (*NC_012920.1:m.15990C>T*) will be added to the database.

References

1. McCormick EM, Lott MT, Dulik MC, et al. Specifications of the ACMG/AMP standards and guidelines for mitochondrial DNA variant interpretation. *Human Mutation*. 2020;41(12):2028-2057. doi:https://doi.org/10.1002/humu.24107

2. Yarham JW, Al-Dosary M, Blakely EL, et al. A comparative analysis approach to determining the pathogenicity of mitochondrial tRNA mutations. *Hum Mutat*. Nov 2011;32(11):1319-25. doi:10.1002/humu.21575

3. MITOMAP. MITOMAP: A Human Mitochondrial Genome Database. Accessed 15 May, 2019. http://www.mitomap.org

4. Brandon MC, Ruiz-Pesini E, Mishmar D, et al. MITOMASTER: a bioinformatics tool for the analysis of mitochondrial DNA sequences. *Human Mutation*. 2009/01/01 2009;30(1):1-6. doi:https://doi.org/10.1002/humu.20801

5. Bianco SD, Parca L, Petrizzelli F, et al. APOGEE 2: multi-layer machine-learning model for the interpretable prediction of mitochondrial missense variants. *Nat Commun*. 2023/08/19 2023;14(1):5058. doi:10.1038/s41467-023-40797-7

6. Sonney S, Leipzig J, Lott MT, et al. Predicting the pathogenicity of novel variants in mitochondrial tRNA with MitoTIP. *PLOS Computational Biology*. 2017;13(12):e1005867. doi:10.1371/journal.pcbi.1005867

7. Landrum MJ, Lee JM, Benson M, et al. ClinVar: public archive of interpretations of clinically relevant variants. *Nucleic Acids Res*. Jan 4 2016;44(D1):D862-8. doi:10.1093/nar/gkv1222

8. Huang G, Wang Y, Yao D. Myoclonic epilepsy with ragged red fibers syndrome associated with mitochondrial 3302A>G mutation in the MT-TL1 gene: A case report. *Exp Ther Med*. Feb 2023;25(2):87. doi:10.3892/etm.2023.11786

9. Santorelli FM, Shanske S, Macaya A, DeVivo DC, DiMauro S. The mutation at nt 8993 of mitochondrial DNA is a common cause of Leigh's syndrome. *Ann Neurol*. Dec 1993;34(6):827-34. doi:10.1002/ana.410340612

10. Yu-Wai-Man P, Griffiths PG, Brown DT, Howell N, Turnbull DM, Chinnery PF. The epidemiology of Leber hereditary optic neuropathy in the North East of England. *Am J Hum Genet*. Feb 2003;72(2):333-9. doi:10.1086/346066

11. Spinazzola A, Carrara F, Mora M, Zeviani M. Mitochondrial myopathy and ophthalmoplegia in a sporadic patient with the 5698G-->A mitochondrial DNA mutation. *Neuromuscul Disord*. Dec 2004;14(12):815-7. doi:10.1016/j.nmd.2004.09.002

12. Olimpio C, Paramonov I, Matalonga L, et al. Increased Diagnostic Yield by Reanalysis of Whole Exome Sequencing Data in Mitochondrial Disease. *J Neuromuscul Dis*. May 13 2024;doi:10.3233/jnd-240020

13. López-Gallardo E, Emperador S, Solano A, et al. Expanding the clinical phenotypes of MT-ATP6 mutations. *Human molecular genetics*. 2014;23(23):6191-6200. doi:10.1093/hmg/ddu339

14. Turro E, Astle WJ, Megy K, et al. Whole-genome sequencing of patients with rare diseases in a national health system. *Nature*. 2020/07/01 2020;583(7814):96-102. doi:10.1038/s41586-020-2434-2

15. Knight KM, Shelkowitz E, Larson AA, et al. The mitochondrial DNA variant m.9032T > C in MT-ATP6 encoding p.(Leu169Pro) causes a complex mitochondrial neurological syndrome. *Mitochondrion*. Nov 2020;55:8-13. doi:10.1016/j.mito.2020.08.009

16. Blakely EL, Yarham JW, Alston CL, et al. Pathogenic mitochondrial tRNA point mutations: nine novel mutations affirm their importance as a cause of mitochondrial disease. *Hum Mutat*. Sep 2013;34(9):1260-8. doi:10.1002/humu.22358

17. Wang J, Balciuniene J, Diaz-Miranda MA, et al. Advanced approach for comprehensive mtDNA genome testing in mitochondrial disease. *Mol Genet Metab*. Jan 2022;135(1):93-101. doi:10.1016/j.ymgme.2021.12.006

18. Mkaouar-Rebai E, Ellouze E, Chamkha I, Kammoun F, Triki C, Fakhfakh F. Molecular-clinical correlation in a family with a novel heteroplasmic Leigh syndrome missense mutation in the mitochondrial cytochrome c oxidase III gene. *J Child Neurol*. Jan 2011;26(1):12-20. doi:10.1177/0883073810371227

19. McCormick EM, Zolkipli-Cunningham Z, Falk MJ. Mitochondrial disease genetics update: recent insights into the molecular diagnosis and expanding phenotype of primary mitochondrial disease. *Curr Opin Pediatr*. 2018;30(6):714-724. doi:10.1097/MOP.0000000000000686
