# Supplementary material for: Mitochondrial DNA disease discovery through evaluation of genotype and phenotype data: The Solve-RD experience
Source: Am J Hum Genet. 2025 Apr 29;112(6):1376–87. doi: 10.1016/j.ajhg.2025.04.003 (PMC12256788; doi:10.1016/j.ajhg.2025.04.003)
Supplement: Document S2. Article plus supplemental information [file mmc4.pdf]

Mitochondrial DNA disease discovery through evaluation of genotype and phenotype data: The Solve-RD experience

Graphical abstract

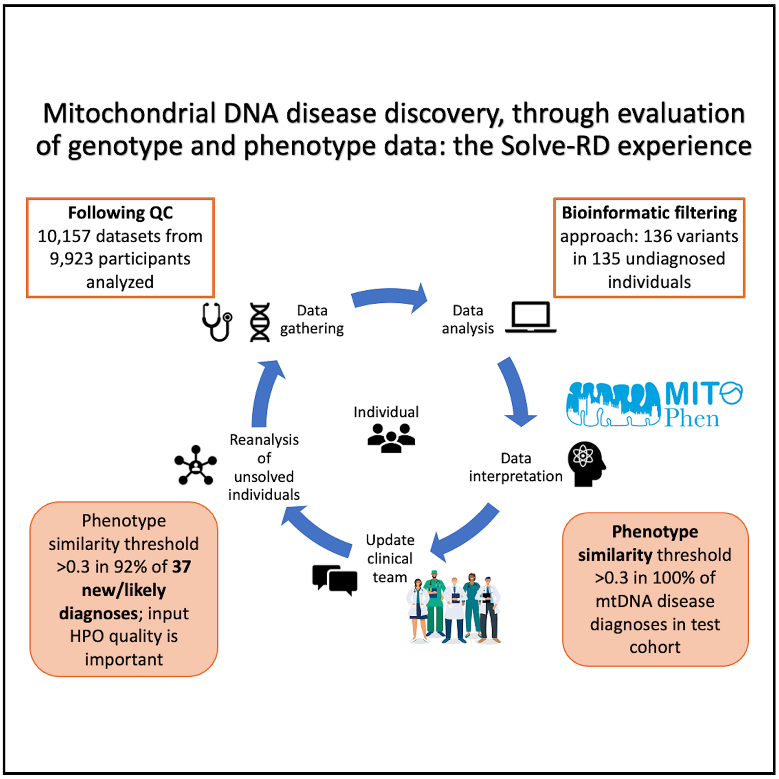

Authors

Thiloka Ratnaike, Ida Paramonov, Catarina Olimpio, ..., Leslie Matalonga, Solve-RD Consortium, Rita Horváth

Correspondence

rh732@medschl.cam.ac.0#uk

Exome and genome reanalysis from the Solve-RD cohort done with an automated mtDNA-filtering pipeline and MitoPhen-based HPO phenotype similarity scoring uncovered previously undiagnosed mtDNA variants, boosting the diagnostic yield by 0.4%. Our structured phenotype evaluation highlights how integrated mtDNA analyses improve rare disease diagnostics, even when mitochondrial conditions are not initially suspected.

Ratnaike et al., 2025, The American Journal of Human Genetics *112*, 1376–1387
June 5, 2025 © 2025 The Author(s). Published by Elsevier Inc. on behalf of American Society of Human Genetics.
https://doi.org/10.1016/j.ajhg.2025.04.003

CellPress

ARTICLE

Mitochondrial DNA disease discovery through evaluation of genotype and phenotype data: The Solve-RD experience

Thiloka Ratnaike,[1,2,6] Ida Paramonov,[3,6] Catarina Olimpio,[4] Alexander Hoischen,[5] Sergi Beltran,[3] Leslie Matalonga,[3] Solve-RD Consortium, and Rita Horváth[4,*]

Summary

The diagnosis of mitochondrial DNA (mtDNA) diseases remains challenging with next-generation sequencing, where bioinformatic analysis is usually more focused on the nuclear genome. We developed a workflow for the evaluation of mtDNA diseases and applied it in a large European rare disease cohort (Solve-RD). A semi-automated bioinformatic pipeline with MToolBox was used to filter the unsolved Solve-RD cohort for rare mtDNA variants after validating this pipeline on exome datasets of 42 individuals previously diagnosed with mtDNA variants. Variants were filtered based on blood heteroplasmy levels ($\geq$1%) and reported association with disease. Overall, 10,157 exome and genome datasets from 9,923 affected individuals from 9,483 families within Solve-RD met the quality inclusion criteria. 136 mtDNA variants in 135 undiagnosed individuals were prioritized using the filtering approach. A focused MitoPhen-based phenotype similarity scoring method was tested in a separate genetically diagnosed "phenotype test cohort" consisting of nuclear gene and mtDNA diseases using a receiving operator characteristic evaluation. We applied the MitoPhen-based phenotype similarity score of >0.3, which was highly sensitive for detecting mtDNA diseases in the phenotype test cohort, to the filtered cohort of 135 undiagnosed individuals. This aided the prioritization of 34 out of 37 (92%) individuals who received confirmed and likely causative mtDNA disease diagnoses. The phenotypic evaluation was limited by the quality of input data in some individuals. The overall pipeline led to an additional diagnostic yield of 0.4% in a cohort where mitochondrial disease was not initially suspected. This highlights the value of our mtDNA analysis pipeline in diverse datasets.

Introduction

The landscape of mitochondrial disease diagnostics has evolved with the establishment of next-generation sequencing (NGS). The diagnostic pathway for these clinically heterogeneous, rare conditions has moved away from the use of muscle biopsy as a first-line test, and blood DNA analysis is increasingly used in the genetic diagnosis of individuals.[1]

At least 1 in 4,300 people are thought to be affected by mitochondrial diseases,[2] and while the prevalence of nuclear mitochondrial diseases may be higher in children, in adults, the majority of individuals present with mitochondrial DNA (mtDNA)-related conditions.[2] Mitochondrial disorders typically have varied clinical presentations at different ages of onset, ranging from rapidly progressive neurodegeneration in early childhood (Leigh syndrome, MIM: 256000) to more slowly progressive adult-onset conditions, such as progressive external ophthalmoplegia and mitochondrial myopathy.[2] The phenotypic spectrum of mtDNA diseases can overlap with common conditions such as diabetes mellitus. An example is the heteroplasmic *MT-TL1* (HGNC: 7490) m.3243A>G (GenBank: NC_012920.1) variant, where some individuals present with diabetes mellitus and hearing loss (maternally inherited diabetes and deafness, MIM: 520000), while other family members can present more severely with mitochondrial encephalopathy, lactic acidosis, and stroke-like episodes (MELAS, MIM: 540000). In addition to the phenotypic heterogeneity, the complexity of interpreting rare mtDNA variants in the diagnostic setting is further compounded by the detection of pathogenic mtDNA variants in individuals not reported to be affected by severe neurological diseases.[3] This may be due to a delayed onset or variable penetrance of clinical features, which may not be captured by databases such as the Genome Aggregation Database. Variant interpretation is challenging due to the lack of clinically validated blood biomarkers,[4] limited availability of additional samples from the proband and matrilineal relatives to confirm segregation of potentially pathogenic mtDNA variants, and the low blood heteroplasmy levels of mtDNA variants historically reported with higher heteroplasmy in post-mitotic tissues, primarily muscle. There are also homoplasmic variants with variable penetrance, such as m.1555A>G (GenBank: NC_012920.1) associated with sensorineural hearing loss, which can be inherited from a homoplasmic clinically unaffected mother. There are currently no established guidelines on

[1]Department of Paediatrics, University of Cambridge, Cambridge, UK; [2]Department of Paediatrics, Colchester Hospital, East Suffolk and North Essex NHS Foundation Trust, Colchester, UK; [3]Centro Nacional de Análisis Genómico (CNAG), Barcelona, Spain; [4]Department of Clinical Neurosciences, School of Clinical Medicine, University of Cambridge, Cambridge, UK; [5]Department of Human Genetics, Radboud University Medical Center, PO Box 9101, 6500 HB Nijmegen, the Netherlands
[6]These authors contributed equally
*Correspondence: rh732@medschl.cam.ac.uk
https://doi.org/10.1016/j.ajhg.2025.04.003.
© 2025 The Author(s). Published by Elsevier Inc. on behalf of American Society of Human Genetics.
This is an open access article under the CC BY license (http://creativecommons.org/licenses/by/4.0/).

how to report back these secondary mtDNA findings to individuals and families.[5]

We have previously developed the MitoPhen database, which captures genotype-phenotype information and pathogenic mtDNA variant heteroplasmy levels in blood and various tissues from published reports.[6] Although its utility through phenotype similarity testing has previously been showcased in a diverse cohort of people with genetic diagnoses, it has not been tested for the prioritization of undiagnosed individuals. Here, we used it to determine sensitive and specific phenotype similarity score thresholds in a dataset of individuals with previously diagnosed mitochondrial diseases caused by heteroplasmic mtDNA variants and those with nuclear gene diagnoses. We then applied this method to search for potentially causative or actionable mtDNA variants within the large European Solve-RD rare disease cohort.

Solve-RD (solving the unsolved rare diseases) is a European-funded research project that investigates individuals with undiagnosed rare diseases who have undergone prior exome (ES) and genome (GS) sequencing with inconclusive results.[7] The aim of the Solve-RD project has been to establish molecular genetic diagnoses for individuals with phenotypically characterized rare diseases recruited through European reference networks (ERNs).[8] Both phenotypic and genetic datasets are submitted to the RD-Connect Genome-Phenome Analysis Platform (GPAP; https://platform.rd-connect.eu/) to aid diagnosis and enable ongoing research efforts.[9] The first step in Solve-RD has been the systematic reanalysis of the previous exome and genome datasets of the unsolved individuals. An international community is involved in the reanalysis efforts within Solve-RD. The Data Analysis Task Force (DATF) and the Data Interpretation Task Force (DITF) work jointly to provide continuous feedback to clinical referrers and researchers. The data are harmonized using the Human Phenotype Ontology (HPO)[10] to record phenotypes, and standardized bioinformatics pipelines are used to annotate genetic variants. Genomic datasets are linked to HPO terms, presenting an opportunity to investigate the quality of the submitted phenotype information and study phenotype similarities between the person's symptoms and the reference datasets.[11] However, given that the HPO was developed primarily using the OMIM database,[12] the phenotypic spectrum of diseases with non-Mendelian inheritance patterns, such as mtDNA diseases, may not be as well covered. Therefore, we wanted to evaluate the utility of a focused phenotype similarity assessment of possible mtDNA diseases, using MitoPhen as the reference database in a large, rare disease cohort without previous systematic analysis of mtDNA variants.

We developed a workflow to identify mtDNA variants using MToolBox,[13] annotated them with the MITOMAP database (http://www.mitomap.org), and subsequently applied sample quality filters and automated variant prioritization. This initial quality control analysis was performed on 11,305 ES and GS datasets, resulting in 10,157 datasets from 9,923 individuals across 9,483 families meeting the quality inclusion criteria. We prioritized mtDNA variants using a heteroplasmy threshold $\geq$1%, reported disease association, and filtered out haplogroup markers—leading to a dataset of 136 variants from 135 individuals. To further stratify the likelihood of a mitochondrial disease diagnosis, we performed phenotype similarity analysis on the 135 prioritized individuals using MitoPhen.[6] We also describe variant pathogenicity assessments in the case of variants of uncertain significance (VUSs) or those not covered in MitoPhen. This approach has enabled a genetic diagnosis in 37 individuals who were previously unsolved by the submitters using their local pipelines to analyze the ES and GS data.

Subjects and methods

Subjects

To verify the accuracy of mtDNA variant calling pipeline, we first analyzed ES data from 42 "pre-solved" individuals (Table S1) with pathogenic heteroplasmic mtDNA variants[13] we diagnosed previously, and ES data were available for analysis. To establish the phenotype similarity threshold, we generated phenotype similarity scores in 47 previously diagnosed individuals with available phenotype information,[6,13] all caused by heteroplasmic mtDNA variants. These 47 previously diagnosed individuals with mtDNA diseases, along with 1,460 individuals diagnosed with nuclear gene disorders in Solve-RD, were included in the phenotypic similarity threshold analysis as a "phenotype test cohort" (Figure 1).

The main study included 10,779 ES and 526 GS datasets from 11,045 affected, undiagnosed individuals submitted by four ERNs: rare neurological diseases (ERN-RND), neuromuscular diseases (ERN-NMD), intellectual disability, telehealth, autism, and congenital anomalies (ERN-ITHACA), and rare and complex epilepsies (ERN-EpiCARE).[7] Clinical data related to symptoms and signs were collected using the HPO format. We excluded datasets from ERN-GENTURIS, which focuses on rare genetic tumor risk syndromes, and ERN-RITA, which investigates rare immunological conditions, as these cohorts were less likely to include mtDNA-related diseases.

The ethics committee/institutional review board (IRB) of University of Tübingen gave ethical approval for this work.

The protocol for the study is found at ClinicalTrials.gov: NCT03491280 (https://clinicaltrials.gov/study/NCT03491280). In compliance with the local ethical guidelines and the Declaration of Helsinki, all individuals (or legal representatives) provided informed consent to participate in the Solve-RD project. All individual-level data were de-identified.

mtDNA variant identification

mtDNA analysis was conducted using MToolBox v.1.2.1.[14] This bioinformatics workflow included mapping reads to the revised Cambridge Reference Sequence (rCRS) and subsequent realignment of reads to the GRCh37/hg19 nuclear genome to discard nuclear mtDNA segments (NUMTs). Following reconstruction of the mitochondrial genome, MToolBox performed variant calling, quantified heteroplasmy, and assigned haplogroups.[14] Haplogroup assignments from mtDNA lineages were linked to geographical locations based on GenBank frequency data.[15] As self-reported ethnicity data

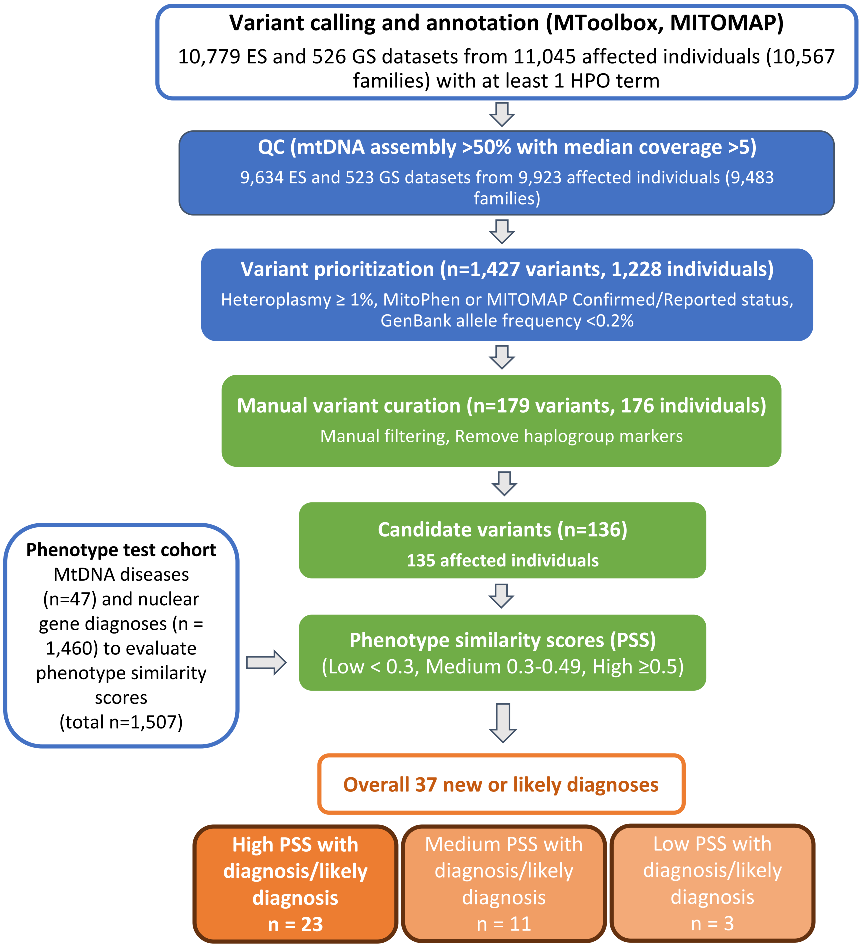

Figure 1. mtDNA variant analysis pathway using data from four ERNs
The numbers listed are the numbers of distinct individual-variant combinations found. Blue and green boxes highlight automated and manual prioritization approaches, respectively, with orange highlighting diagnostic/likely causative mtDNA variants. There were 135 individuals with 136 variants found after MITOMAP, MToolbox, and manual filtering approaches. The phenotype similarity score evaluation utilized results from the phenotype test cohort, which included the 47 previously diagnosed individuals with mtDNA diseases (total $n = 1{,}507$). The thresholds were applied to evaluate the 135 undiagnosed individuals with 136 rare mtDNA variants, and the numbers of individuals with new or likely mtDNA disease diagnoses are shown. HPO, Human Phenotype Ontology term; mtDNA, mitochondrial DNA; PSS, phenotype similarity score; QC, quality control.

were not collected, this step enabled a characterization of the cohort in terms of mtDNA genetic ancestry in order to understand the diversity of the cohort. This also enabled us to filter out mtDNA variants that were likely polymorphisms associated with haplogroups.

Validation of the pipeline in a pre-solved cohort

mtDNA sequencing of the pre-solved individuals has been performed by both sequencing (Sanger sequencing and, for some variants, also by pyrosequencing) and ES. Comparison of mtDNA heteroplasmy determined by these alternative methods showed a good correlation.[13] mtDNA assembly and coverage were assessed in each sample, as well as the heteroplasmy levels of the identified variants. This cohort was only included to validate our bioinformatic and phenotype similarity analyses (see below) but was not included in the overall diagnostic yield results.

mtDNA analysis in the unsolved cohort

Overall, 11,305 datasets were analyzed, including 10,779 ES and 526 GS datasets from 11,045 affected individuals. There were 234 individuals with two datasets, 10 individuals with three datasets, and 2 individuals with four datasets; the remaining 10,799 individuals had one dataset each. Most DNA samples (11,166/11,305, 98.8%) were derived from blood, 9 were from muscle, and the other 130 samples were from 11 different tissues. Genomic data from the ES datasets were generated using 30 different enrichment kits. Only one kit (Nimblegen_SeqCapEZMedExomePlus MT_47Mb), used in 359/10,779 (3.3%) of ES datasets, allowed for direct target enrichment of the mitochondrial genome, whereas in the others, mtDNA sequences were obtained through off-capture sequencing. Only datasets in which at least 50% of the mtDNA was assembled and with at least 5× coverage were considered for further analysis. mtDNA variants were annotated with information from MITOMAP and MitoPhen databases and prioritized based on the following criteria: (1) heteroplasmy level ≥ 1%, (2) "confirmed" disease association in MITOMAP, (3) disease association in MitoPhen, or (4) "reported" disease-association in MITOMAP with allele frequency in MITOMAP < 0.2%. Phenotype similarity scores were generated as previously published,[6] and HPO data per individual were used to annotate the variants.

Phenotype similarity analysis

Phenotype similarity evaluation was undertaken using OntologyX,[16] and results were visualized using ggplot2[17] and gplots[18] R packages. HPO-based phenotype similarity scores between probands in MitoPhen and affected participants were computed as previously described using Lin's measure of similarity.[6]

To gather available evidence for relevant phenotype similarity thresholds before the annotation of the Solve-RD dataset, we used the phenotype test cohort, which included data from 47 diagnosed individuals with heteroplasmic mtDNA variants from the pre-solved cohort and our previous publication.[6] We separately performed an analysis that included all individuals from the pre-solved cohort and previous publication ($n = 119$). This meant that individuals with and without variants known to cause Leber hereditary optic neuropathy (LHON; MIM: 535000),[19] such as m.11778G>A (GenBank: NC_012920.1), m.14484T>C (GenBank: NC_012920.1), and m.3460G>A (GenBank: NC_012920.1), and variants associated with sensorineural hearing loss, which can be caused by aminoglycoside use,[20] such as m.1494C>T (GenBank: NC_012920.1) and m.1555A>G (GenBank: NC_012920.1), were analyzed. This was because these individuals would

have higher phenotype similarity scores overall due to the associated narrow phenotypic spectrums of the variants. We measured the performance of the phenotype similarity score predictor in the previously diagnosed individuals by comparing these with phenotype similarity scores generated on 1,460 persons with a confirmed nuclear genetic diagnosis within Solve-RD. The pROC R package[21] was used to conduct the receiving operator characteristic (ROC) analysis. The area under the curve (AUC) was used to evaluate the discriminatory performance of phenotype similarity in distinguishing the diagnosis type.[22] Specificity and sensitivity of achieving mtDNA disease diagnoses were analyzed using the phenotype test cohort. We grouped the individuals with nuclear genetic diagnoses as "nuclear-mitochondrial" or "nuclear-other" based on PanelApp[23] (mitochondrial disease gene panels or not) to understand whether individuals with nuclear-mitochondrial disease had higher phenotype similarity than nuclear-other genetic diagnoses. Youden's index[24] was used to determine the optimal threshold for sensitivity and specificity of the predictor variable. We established a phenotype similarity score for a highly sensitive pick-up rate of mtDNA diseases and used this threshold to set arbitrary thresholds for the phenotype similarity groups: "low," "medium," and "high."

Manual mtDNA variant curation in the unsolved cohort

Candidate mtDNA variants were prioritized utilizing phenotype similarity and discussed with an expert in mitochondrial disease (R.H.) and a clinical geneticist (C.O.). All variants were evaluated using ACMG criteria[25] (supplemental methods), and prioritized variants were submitted according to the likelihood of causing disease to the Solve-RD DITF.

Clinical feedback

"Diagnostic" (used synonymously with "causative") variants were those where the diagnosis was confirmed by the recruiting clinician. "Likely causative" variants for the individual's phenotype were those that were submitted to the DITF, but we have not had final confirmation from the recruiting clinician. We also submitted "reportable findings," where partial HPO matches were present and our team decided that these variants may be causative for the symptoms or relevant to discuss with the recruiting clinicians in case genetic counseling is warranted. These included homoplasmic or near-homoplasmic variants with variable penetrance in the population but with potential clinically actionable consequences—such as variants that can cause LHON[19] or variants such as m.1555A>G (NC_012920.1) associated with sensorineural hearing loss, which can be caused by aminoglycoside use.[20]

Results

mtDNA variant identification and validation of the pre-solved cohort

We were able to identify the causative variant in all 42 pre-solved individuals, with heteroplasmy levels ranging from 33 to 100% and with phenotype similarity scores > 0.3. Heteroplasmy levels were consistent across the different methods (Sanger sequencing, pyrosequencing, and ES).[13] mtDNA assembly varied from 62% to 100%, with a minimum of 7× and a maximum of 165× chrM coverage depth (Table S1). Based on these results, a minimum quality threshold was set at 50% assembly and 5× coverage to allow for the high variability across different exome kits (Figure S1).

mtDNA analysis in the unsolved cohort

From the 11,305 datasets analyzed in the overall unsolved cohort, 10,157 (90%) met our quality inclusion criteria (Figure S2). In total, 523/526 (99%) of GS and 9,634/10,557 (89%) of ES datasets were included in further analysis (Figure S3). These were samples from 9,923 affected individuals belonging to 9,483 families. We applied the MToolBox[14] bioinformatics pipeline alongside our automated prioritization approach, which led to 179 variants in 176 individuals for the initial manual assessment to exclude haplogroup markers and likely benign variants (Figure 1; supplemental results; Figure S4). This manual analysis prioritized 135 individuals with 136 variants for further clinical evaluation. 130 samples (96%) belonged to mtDNA lineages associated with European ancestry (haplogroups H, J, K, T, and U). Of these 135 individuals, we have established mtDNA disease diagnosis in 21 individuals, while 16 had likely causative variants (Figure 1; Table S2). Notably, 17 of the 21 disease-causing variants were detected in ES data (with 1 found at <10× mtDNA coverage), while 4 were found in GS (Figure S3). All 16 likely causative variants were identified using ES. In 10 of the 135 individuals, a nuclear disease gene had been identified as the genetic diagnosis on Solve-RD GPAP, and the mtDNA variants in these individuals were not thought to contribute to the phenotype.

Phenotype similarity analysis

Phenotype similarity scores were conducted using MitoPhen in the phenotype test cohort: 47 individuals with previous mtDNA disease diagnoses and 1,460 individuals with a nuclear genetic diagnosis. In 122 individuals, a nuclear gene diagnosis was associated with mitochondrial disease (nuclear-mitochondrial), while in 1,338, the diagnosis involved other nuclear genes (nuclear-other). The phenotype similarity data are shown in Figure 2A; a higher mean phenotype similarity score was observed in individuals with mtDNA diseases ($n = 47$) compared with both groups of nuclear gene diagnoses ($p < 0.0001$). Further evaluation of the optimal phenotype similarity score for mtDNA diseases using Youden's index[24] found that a score of 0.48 achieved a sensitivity of 94% and a specificity of 65% in correctly predicting mtDNA disease over nuclear gene diseases, whereas a threshold of 0.5 achieved a lower sensitivity of 74% but a higher specificity of 71%. The phenotype similarity threshold of 0.3 achieved a sensitivity of 100% but a low specificity of 15% (Figure 2B). The ROC analysis generated an AUC of 0.82 (95% confidence interval [CI]: 0.76–0.88), indicating good discriminatory power[26] of phenotype similarity scores for distinguishing individuals with mtDNA diseases from those with

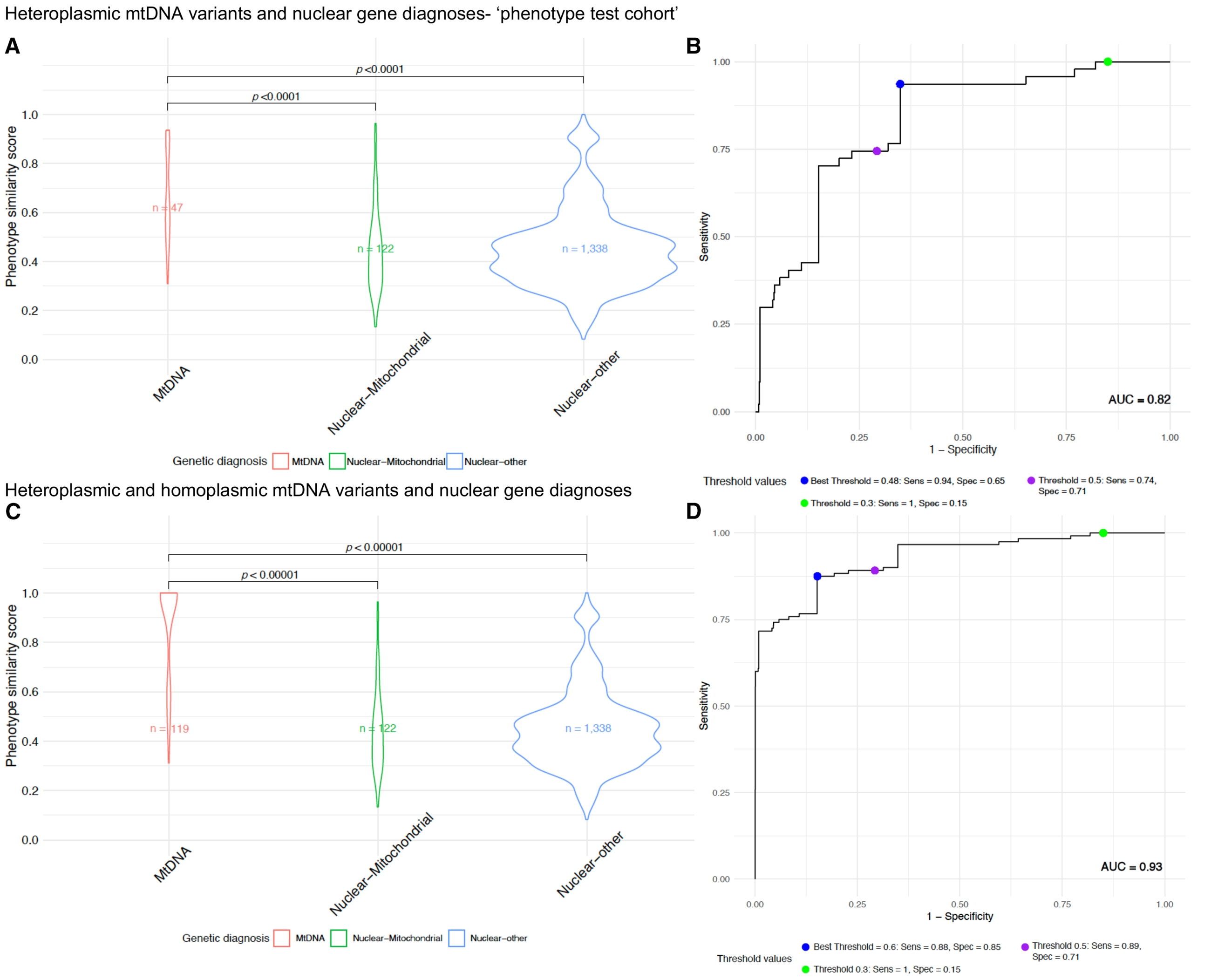

Figure 2. Evaluation of phenotype similarity score thresholds with diagnosed individuals
(A) Phenotype similarity scores between previously diagnosed individuals with heteroplasmic mitochondrial DNA (mtDNA) variants and nuclear-mitochondrial and nuclear-other genetic diagnoses. The mtDNA disease group overall had significantly higher overall phenotype similarity values as computed using the Kruskal-Wallis test followed by Dunn's post hoc test with Bonferroni correction for multiple groups.[17]
(B) Receiver operator curve characteristic calculation for the diagnostic performance of phenotype similarity scores, using pROC,[21] achieved an area under the curve (AUC) value of 0.82. The phenotype similarity threshold of 0.48 (blue) was determined using Youden's index, and the rounded threshold of 0.5 (purple) is also shown, which optimizes the balance between sensitivity and specificity. However, a threshold of 0.3 (green) achieved 100% sensitivity.
(C) Phenotype similarity scores between diagnosed individuals with heteroplasmic and homoplasmic mtDNA variants and nuclear-mitochondrial and nuclear-other genetic diagnoses. The mtDNA disease group showed significantly higher overall phenotype similarity values as computed using the Kruskal-Wallis test followed by Dunn's post hoc test with Bonferroni correction for multiple groups.[17]
(D) Receiver operator curve characteristic calculation achieved an AUC value of 0.93. The phenotype similarity threshold of 0.6 (blue) was determined using Youden's index, and the thresholds of 0.5 (purple) and 0.3 (green) are shown. Sens, sensitivity; Spec, specificity.

nuclear genetic diseases. We, therefore, arbitrarily set our phenotype similarity threshold below 0.3 as “low phenotype similarity” and 0.5 or greater as “high phenotype similarity” for subsequent analyses. Furthermore, we analyzed the phenotype test cohort in addition to diagnosed individuals with mtDNA variants known to cause LHON and sensorineural hearing loss ($n = 119$) and found highly significant differences between the mtDNA and nuclear genotypes based on phenotype similarity scores, with a higher AUC of 0.93 using ROC analysis (Figures 2C and 2D).

The phenotypic spectrum was assessed using HPO terms. The mtDNA disease group had more multisystem presentations compared to those with nuclear genetic diagnoses, which explained the differences seen in phenotype similarity scores (supplemental results; Figure S5).

Manual mtDNA variant curation in the unsolved cohort

This section discusses the evaluation of 136 rare mtDNA variants in 135 undiagnosed individuals using phenotype similarity scores. The actions taken for the unsolved cohort

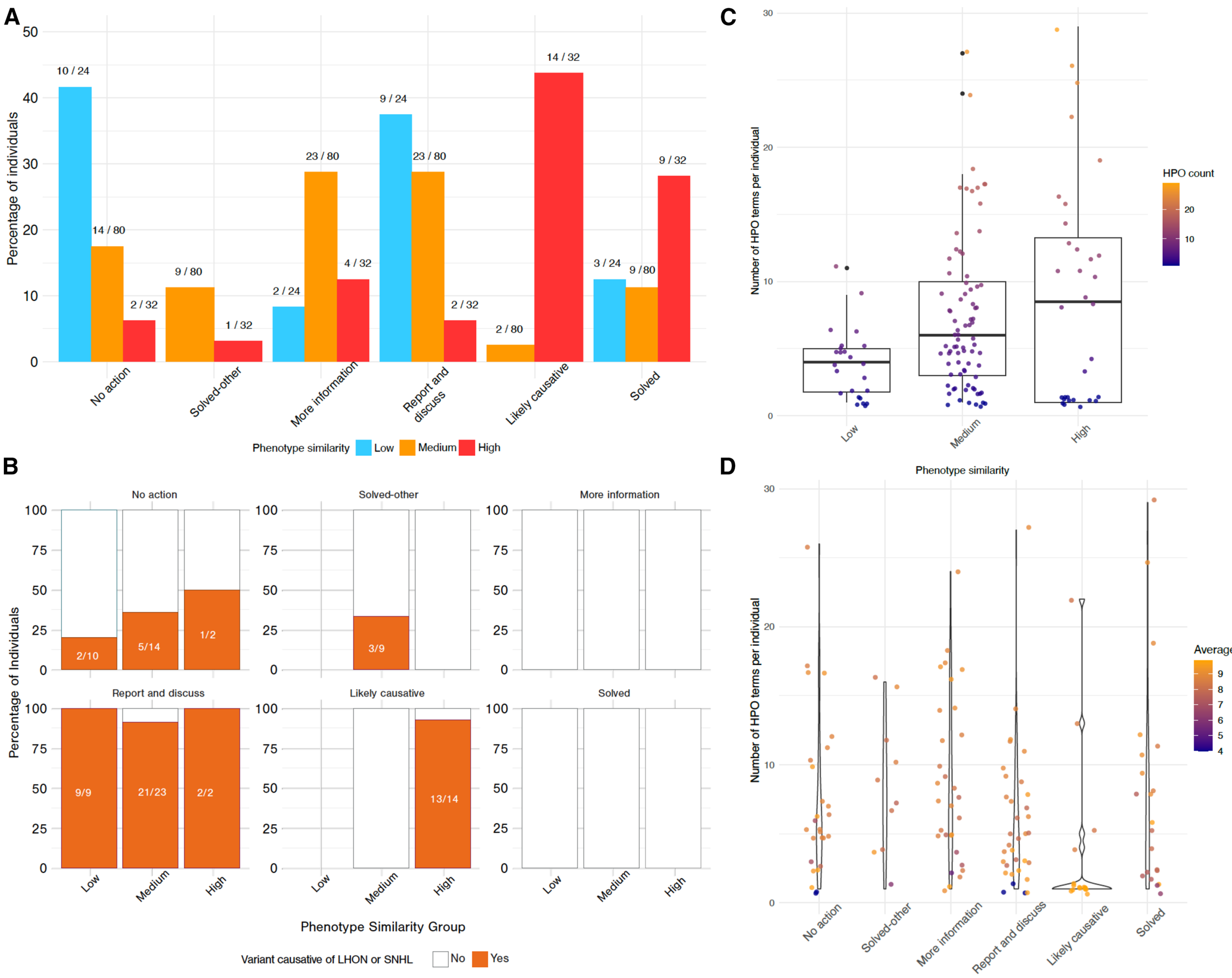

Figure 3. The actions taken for the previously undiagnosed group

(A) Action and phenotype similarity groups for the mtDNA variants: blue highlights low phenotype similarity (<0.3), orange shows the medium scores (≥0.3 < 0.5), and red shows high scores (≥0.5). The count of individuals per phenotype similarity group is given as the denominator, and the person count per action is given as the nominator.

(B) Faceted plot per "action" grouping, showing phenotype similarity group on the x axis, and the percentage of individuals with a mtDNA variant known to cause Leber's hereditary optic neuropathy (LHON) or sensorineural hearing loss (SNHL) shown in orange; the number of individuals is shown within the bars. The "repeat and discuss" group contained the largest relative proportions of these variants, as did the high phenotype similarity group with likely causative variants.

(C) Human Phenotype Ontology (HPO) terms collected per individual and the phenotype similarity grouping.

(D) Violin plot displays the HPO count data per person, grouped by overall action taken, and the points are colored by the average information content (IC) per person using non-redundant HPO terms.

($n = 135$) are shown in Figure 3A. We detected high phenotype similarity scores in 32 of the 136 mtDNA variants; 23 (72%) were diagnostic or likely causative, and one (3%) was solved with the nuclear gene *WAC* (HGNC: 17327). Two out of the 32 variants (6%) were categorized as "no further action needed" (homoplasmic, likely benign m.14696A>G [GenBank: NC_012920.1] and m.1555A>G [GenBank: NC_012920.1] at 62% heteroplasmy). Another two individuals (6%) had reportable findings (homoplasmic m.1555A>G [GenBank: NC_012920.1]), and more information was needed in terms of variant segregation and phenotype for the remaining four (13%): m.3243A>G (GenBank: NC_012920.1), m.3249G>A (GenBank: NC_012920.1), m.4308G>A (GenBank: NC_012920.1), and m.16023G>A (GenBank: NC_012920.1) (Table S3). LHON or sensorineural-hearing-loss-associated variants were detected in 12 individuals with high phenotype similarity scores, aiding diagnosis (Figure 3B). There were 80/136 variants (59%) with medium phenotype similarity scores; careful analysis confirmed that 11 of these (14%) were causative or likely causative variants for the clinical presentations. Most variants (46/80, 58%) with medium phenotype scores were actionable, as either further information was requested from the recruiting clinicians or genetic counseling was recommended if homoplasmic variants known to be associated with LHON or sensorineural hearing impairment were detected. Overall, 34/37 (92%) of individuals with diagnosed or likely causative variants had a phenotype similarity score > 0.3. Only 3 individuals with low phenotype similarity scores (range: 0.21–0.29) carried

Table 1. Number of affected individuals in whom at least 50% of the mtDNA had 5× coverage and with HPO data available

ERN	Total experiments	No. of individuals	No. of families	Individuals solved with mt variant	Candidate mt variant	Diagnostic yield % = solved proband/no. of families
ERN-ITHACA	4,024	3,883	3,783	3	2	0.08
ERN-RND	3,121	3,052	2,880	11 (10 probands)	0	0.35
ERN-EURO-NMD	1,707	1,683	1,534	7 (6 probands)	13	0.39
ERN-EpiCARE	1,305	1,305	1,286	0	1	0
Total	10,157	9,923	9,483	21 (19 probands)	16	0.20–0.37[a]

The number of samples, families, and persons with mtDNA diagnoses/candidate variants are given by European reference network (ERN).
[a]The diagnostic yields without and with the inclusion of likely causative variants are 0.20% and 0.37%, respectively.

well-established pathogenic mtDNA variants in high heteroplasmy levels (>90%) (Table S2). Two of these individuals had only one HPO term provided, and the third one had three non-redundant terms. Therefore, the number and the information content (IC) of HPO terms were likely to lead to low phenotype similarity in these persons (Figures 3C and 3D). Dunn's test with Bonferroni correction[27,28] revealed significant differences between phenotype similarity groups and HPO counts, high versus low ($p = 0.0296$) and medium versus low ($p = 0.0086$), indicating increased HPO term counts were associated with higher phenotype similarity scores. There was a spread of the average IC per person, with a trend toward higher average IC seen in those with likely causative or diagnostic variants. The differences in average IC were significant between the likely causative group and other groups ($p < 0.05$, using Dunn's test and Bonferroni correction) (Figure 3D). This highlighted that more specific HPO terms were associated with a better diagnostic yield of causative mtDNA variants using phenotype similarity scores.

All individuals with likely causative and diagnostic mtDNA variants had blood heteroplasmy levels > 11%, and most of the diagnoses were in individuals recruited through ERN-RND ($n = 11$) (Figure S6). The overall diagnostic rate for mtDNA diseases across the 9,923 affected individuals from 9,483 families was 0.4%, with 37 new confirmed and likely diagnoses (Table 1).

Variants not in MitoPhen

The overall mtDNA analysis pipeline highlighted 26 rare VUSs not included in the MitoPhen database across 29 individuals (P19–P21 and P38–63; Table S2). We investigated the pathogenicity of these 26 mtDNA variants using the ACMG guidelines for the interpretation of mtDNA variants.[25] All 26 variants were previously reported in the literature with a possible association with mitochondrial disease, but previous data were not stringent enough to include these in the MitoPhen database. Six of the 26 variants had a high phenotype similarity score (≥0.5), and in 2 out of 6 (33%), it supported the genetic diagnosis (m.5698G>A [GenBank: NC_012920.1][29] and m.9032T>C [GenBank: NC_012920.1]), albeit the ACMG criteria did not meet likely pathogenic status (P19 and P20). Another individual (P21) who carried the homoplasmic m.9478T>C (NC_012920.1) variant (classified as a VUS) associated with a medium phenotype similarity score (0.33) was classified by the local laboratory as pathogenic. Only P42 had a variant classified as likely pathogenic, m.15990C>T (NC_012920.1), with a low heteroplasmy level (4%); however, they had an alternative nuclear genetic diagnosis on GPAP. Further details about these variants are found in the supplemental note (Table S2).

Discussion

Diagnosing mtDNA-related disease is still challenging in the era of first-line genomic testing. Some well-established pathogenic mtDNA variants can have low heteroplasmy in blood (e.g., m.3243A>G [GenBank: NC_012920.1]),[30] leading to uncertainty about the correlation with clinical features, while others are homoplasmic but with low penetrance.[31] To facilitate the detection of pathogenic mtDNA variants in the Solve-RD project, we have applied a semi-automated workflow that enabled a large-scale reanalysis of ES and GS data for possible mtDNA diseases applying a new HPO-based method using phenotype similarity scores with previously reported individuals. We evaluated mtDNA variants in this large heterogeneous rare disease cohort following quality control filters in 9,923 individuals from 9,483 families and identified 135 individuals with potentially relevant mtDNA variants for further analysis. We have generated confirmed and likely diagnoses in 37 individuals, leading to an overall 0.4% diagnostic yield in 9,483 families, which is reflective of the rarity of these conditions. Among individuals with ES data, 17 had confirmed diagnoses, and 16 had likely diagnoses, resulting in a diagnostic yield of 0.36% (33 probands/9,233 families). However, when considering individuals diagnosed solely based on GS data (2 probands/467 families), the diagnostic yield was 0.43%, which was lower than the yield for suspected mitochondrial diseases investigated in the 100K Genome Project in the UK, which was 6/319 (2%).[32] Our cohort was not suspected of having primarily mitochondrial diseases; it was a less selective and more phenotypically heterogeneous cohort, likely explaining the lower diagnostic yield. This is, however, in

keeping with the diagnostic yield in a similar cohort collated through the Genomics Research to Elucidate the Genetics of Rare Diseases Consortium (GREGoR).[33] In the GREGoR study, the authors used the mitochondrial disease criteria (MDC) score,[34] adapted for HPO terms, to consider the likelihood of a mitochondrial disease explaining the person's phenotype.[33] The authors identified 10 new mtDNA diagnoses, 1 novel *POLG* (OMIM: 174763) variant (with several mtDNA variants), and 11 candidate diagnoses, resulting in a diagnostic uplift of 0.4% (22/5,625) in undiagnosed families. In our study, comprehensive phenotype analysis using HPO terms enabled us to prioritize 0.4% confirmed mtDNA variants efficiently in a similarly heterogeneous cohort where mitochondrial disease was previously not suspected. We also recently published our analysis of mtDNA variants from ES data in persons with neuromuscular conditions from South Africa, Brazil, India, Turkey, and Zambia.[34] In 998 individuals, there were two definite diagnoses, two possible diagnoses, and eight secondary findings. Surprisingly, common pathogenic mtDNA variants found in people of European ancestry were very rare, and the landscape of pathogenic mtDNA variants differs around the world. The reanalysis of 56,434 GS in the gnomAD v.3.1 database in various populations detected that 1/250 individuals carry a pathogenic mtDNA variant with heteroplasmy above 10%, who were not known to have significant neurological disorders.[3] Therefore, a pipeline that enhances the reanalysis of ES and GS with a structured phenotype similarity evaluation is timely to aid the interpretation of these mtDNA variants.

One of the complexities when using NGS data that does not specifically target mtDNA is achieving a sufficient depth of coverage of the mitochondrial genome. While most GS data used in this study provided high median mtDNA coverage (2,347×), sequence data derived from ES was highly variable across different exome kits and even across samples sequenced using the same kit (Figure S1). Based on the successful identification of pathogenic mtDNA variants in our validation set of pre-solved individuals with low mtDNA coverage (7×), we decided to include exomes from the unsolved cohort for the analysis. Overall, a median mtDNA read depth in ES (34×) was sufficient to keep 89% of exome datasets for further investigation. Another important challenge when using ES and GS to interrogate mtDNA is the co-amplification of NUMTs.[35] The MToolBox pipeline[14] includes a process to filter out such sequences to minimize the occurrence of false positive variants.

Our results suggest that incorporating precise mtDNA analysis into routine ES data analysis pipelines has a role in the genetic diagnosis of these rare diseases. These analyses were computationally efficient due to the small size of the mitochondrial genome (16.5 kb) and could be largely automated without creating a significant interpretation burden. This is well demonstrated by the fact that our pipeline enabled us to restrict the number of possible mtDNA variants to only 179 variants (in 176 individuals) from 9,923 individuals (2%) who passed quality control steps (Figure 1). Nonetheless, based on the technical challenges highlighted above, an additional validation of candidate variants using alternative methods is highly recommended.[5]

In our study, 96% of the samples included in genotype-phenotype analyses belonged to Eurasian mtDNA lineages, mainly from European genetic ancestry, according to haplogroup analyses (Figure S4). Therefore, similar work in geographically and ethnically diverse datasets would be useful to confirm that our pipeline is also applicable in other populations. mtDNA haplogroup analysis helped us exclude 43 variants that had previous conflicting evidence of pathogenicity; therefore, we suggest including this step in bioinformatic pipelines of mtDNA analysis.[36,37] Further work should incorporate the use of mtDNA allele frequencies from gnomAD to reflect likely population prevalence.[3]

We applied a phenotype-driven approach based on phenotype similarity thresholds, following a ROC analysis to determine the sensitivities and specificities of the genetic diagnoses. This method was able to detect previously confirmed pathogenic mtDNA variants in ES and GS datasets and led to new diagnoses within a large unsolved cohort by applying a novel phenotype similarity score-based analysis. We found that the phenotype similarity thresholds were useful in prioritizing variants because 92% of individuals with diagnosed or likely causative variants had a phenotype similarity score > 0.3. There were no likely causative variants found with a low phenotype similarity (<0.3). Therefore, although the threshold of 0.3 has a low specificity, it has a high sensitivity and can prioritize variants for manual curation. We also applied this threshold for variants not included in MitoPhen, demonstrating that a phenotype similarity filter can be a valid approach to facilitate mtDNA diagnoses in large heterogeneous cohorts. However, our data suggest that participants with limited or non-specific HPO terms are less likely to receive diagnoses and can limit the utility of our focused phenotype-based approach. In support of this observation, previous work has shown that five or more HPO terms are required for the optimal identification of the genetic diagnosis.[11] Therefore, we would recommend using similar minimal phenotypic data standards in future studies.

We noted that all pathogenic and likely causative variants were found in blood at heteroplasmies of >11%. Participants have been diagnosed with lower blood heteroplasmy levels using GS[38]; however, it becomes difficult to confidently diagnose individuals without further information on heteroplasmy in other tissues or segregation within the family.

The current pipeline suggests that following careful genotype filtering, incorporating a mtDNA disease-focused phenotype similarity approach can be effective in highlighting potential pathogenic mtDNA variants. Our

pipelines will need to be validated in more clinically and ethnically diverse cohorts that include a broad spectrum of diseases. This model of iterative genotype-phenotype analysis incorporating HPO-based curated databases such as MitoPhen will enable streamlined diagnostic pathways and tailored counseling for individuals with rare diseases and their families.

Conclusion

We have demonstrated an effective strategy to detect relevant mtDNA variants in ES or GS datasets. We used HPO data to facilitate the diagnosis and prioritization of mtDNA diseases within Solve-RD, where mitochondrial disease was not the primary suspected diagnosis. We have established that incorporating a structured phenotype evaluation with similarity score thresholds is likely to increase the yield of new diagnoses and can be a useful filtering approach in the analysis of large, rare disease cohorts. However, it is important to acknowledge that individuals with low heteroplasmic or rare mtDNA variants with limited HPO data should be carefully considered for ongoing multidisciplinary discussions, and feedback from the expert clinical team is vital to support the diagnosis.

Data and code availability

This study did not generate new datasets. The published article by Laurie et al. includes all datasets analyzed during this study.[39]

Consortia

The members of the Solve-RD consortium are Olaf Riess, Tobias B. Haack, Holm Graessner, Birte Zurek, Kornelia Ellwanger, Stephan Ossowski, German Demidov, Marc Sturm, Joohyun Park, Leon Schütz, Julia M. Schulze-Hentrich, Rebecca Schüle, Jishu Xu, Melanie Kellner, Baptist Resch, Ingrid Kolen, Matthis Synofzik, Carlo Wilke, Andreas Traschütz, Danique Beijer, Peter Heutink, Ludger Schöls, Holger Hengel, Holger Lerche, Christian Boβelmann, Josua Kegele, Robert Lauerer-Braun, Stephan Lauxmann, Han Brunner, Hans Scheffer, Nicoline Hoogerbrugge, Alexander Hoischen, Peter A.C. 't Hoen, Lisenka E. L.M. Vissers, Christian Gilissen, Wouter Steyaert, Karolis Sablauskas, Richarda M. de Voer, Erik-Jan Kamsteeg, Bart van de Warrenburg, Nienke van Os, Iris te Paske, Erik Janssen, Elke de Boer, Marloes Steehouwer, Burcu Yaldiz, Kornelia Neveling, Bart van der Sanden, Lydia Sagath, Tjitske Kleefstra, Anthony J. Brookes, Spencer Gibson, Umar Riaz, Greg Warren, Sai Anuhya Nalagandla, Yunze Patrick Wang, Deepthi Sukumaran, Sadegh Abadijou, Ana Töpf, Volker Straub, Chiara Marini Bettolo, Jordi Diaz Manera, Sophie Hambleton, Karin Engelhardt, Jill Clayton-Smith, Siddharth Banka, Elizabeth Alexander, Adam Jackson, Laurence Faivre, Christel Thauvin, Antonio Vitobello, Anne-Sophie Denommé-Pichon, Yannis Duffourd, Ange-Line Bruel, Victor Couturier, Sergi Beltran, Ivo Glynne Gut, Steven Laurie, Davide Piscia, Leslie Matalonga, Anastasios Papakonstantinou, Gemma Bullich, Alberto Corvo, Marcos Fernandez-Callejo, Carles Hernández, Daniel Picó, Ida Paramonov, Anna Esteve Codina, Marc Dabad, Marta Gut, Emanuele Raineri, Hanns Lochmüller, Gulcin Gumus, Virginie Bros-Facer, Ana Rath, Marc Hanauer, David Lagorce, Oscar Hongnat, Maroua Chahdil, Caterina Lucano, Emeline Lebreton, Giovanni Stevanin, Alexandra Durr, Claire-Sophie Davoine, Léna Guillot-Noel, Anna Heinzmann, Giulia Coarelli, Gisèle Bonne, Teresinha Evangelista, Valérie Allamand, Isabelle Nelson, Rabah Ben Yaou, Corinne Metay, Bruno Eymard, Enzo Cohen, Antonio Atalaia, Tanya Stojkovic, Milan Macek Jr., Marek Turnovec, Dana Thomasová, Radka Pourová Kremliková, Vera Franková, Markéta Havlovicová, Lukáš Ryba, Petra Lišková, Pavla Doležalová, Alice Krebsová, Helen Parkinson, Thomas Keane, Mallory Freeberg, Coline Thomas, Dylan Spalding, Peter Robinson, Daniel Danis, Glenn Robert, Alessia Costa, Mike Hanna, Henry Houlden, Mary Reilly, Jana Vandrovcova, Stephanie Efthymiou, Heba Morsy, Elisa Cali, Francesca Magrinelli, Sanjay M. Sisodiya, Ravishankara Bellampalli, Patrick Moloney, Jonathan Rohrer, Francesco Muntoni, Irina Zaharieva, Anna Sarkozy, Luke Perry, Veronica Pini, Juliane Müller, Vincent Timmerman, Jonathan Baets, Geert de Vries, Jonathan De Winter, Peter de Jonghe, Liedewei Van de Vondel, Willem De Ridder, Sarah Weckhuysen, Hannah Stamberger, Charissa Millevert, Noor Smal, Vincenzo Nigro, Manuela Morleo, Michele Pinelli, Sandro Banfi, Annalaura Torella, Roberta Zeuli, Mariateresa Zanobio, Giulio Piluso, Alessandra Ferlini, Rita Selvatici, Francesca Gualandi, Stefania Bigoni, Marcella Neri, Stefan Aretz, Isabel Spier, Anna Katharina Sommer, Sophia Peters, Carla Oliveira, Jose Garcia-Pelaez, Rita Barbosa-Matos, Celina São José, Marta Ferreira, Irene Gullo, Susana Fernandes, Luzia Garrido, Pedro Ferreira, Fátima Carneiro, Morris A Swertz, Lennart Johansson, Joeri K van der Velde, Gerben van der Vries, Pieter B Neerincx, Dieuwke Roelofs-Prins, David Ruvolo, Marielle van Gijn, Kristin M Abbott, Wilhemina S. Kerstjens Frederikse, Eveline Zonneveld-Huijssoon, Sebastian Köhler, Alison Metcalfe, Richard Moore, Alain Verloes, Séverine Drunat, Delphine Heron, Cyril Mignot, Boris Keren, Jean-Madeleine de Sainte Agathe, Rami Abou Jamra, Marc Abramowicz, Özge Aksel Kiliçarslan, Nicholas Allen, Francisco Javier Alonso García de la Rosa, Simona Balestrini, Peter Balicza, Tobias Bartolomaeus, Ayşe Nazlı Başak, Laura Batlle Masó, David Beeson, Valerie Benoit, Katherine Benson, Eva Bermejo Sánchez, Emilia K. Bijlsma, Elke Bogaert, Mara Bourbouli, Kaan Boztug, Sylvain Brohée, Susan Byrne, Andrés Caballero Garcia de Oteyza, Gabriel Capella, Evelina Carpancea, Gianpiero Cavalleri, Ana Cazurro-Gutiérrez, Patrick F. Chinnery, Maria-Roberta Cilio, Andrea Ciolfi, Kristl Claeys, Roger Colobran, Isabell Cordts, Judith Cossins, Karin Dahan, Bruno Dallapiccola, Norman Delanty, Christel Depienne, Chantal Depondt, Bart Dermaut, Marcus Deschauer, Julie

Desir, Anne Destrée, Minas Drakos, Sarah Duerinckx, Berta Estevez, Athanasios Evangeliou, Chiara Fallerini, Marco Ferilli, Simone Furini, Julien Gagneur, Hamidah Ghani, Marie Greally, Bodo Grimbacher, Renzo Guerrini, Peter Hackman, Matthias Haimel, Eva Hammar Bouveret, Dimitri Hemelsoet, Rebecca Herzog, Mariette J.V. Hoffer, Elke Holinski-Feder, Rita Horvath (ORCID), Manon Huibers, Michele Iacomino, Mridul Johari, Elisabeth Kapaki, Deniz Karadurmus, Mert Karakaya, Evgenia Kokosali, Christian Korff, Leon Krass, Didier Lacombe, Andreas Laner, Helen Leavis, Damien Lederer, Elsa Leitão, Hanns Lochmüller, Katja Lohmann, Estrella López Martín, Rebeka Luknárová, Alfons Macaya, Sivasankar Malaichamy, Anna Marcé-Grau, Beatriz Martínez Delgado, Sandrine Mary, Frédéric Masclaux, Lambros Mathioudakis, Ales Maver, Patrick May, Isabelle Maystadt, Davide Mei, Christian Mertes, Colombine Meunier, Maria Judit Molnar, Olivier Monestier, Stéphanie Moortgat, Alexander Münchau, Francina Munell, Andrés Nascimento Osorio, Daniel Natera de Benito, Mary O Reghan, Catarina Olimpio, Elena Parrini, Martje Pauly, Belén Pérez-Dueñas, Borut Peterlin, Konrad Platzer, Kiran Polavarapu, Bruce Poppe, Manuel Posada De la Paz, Flavia Privitera, Francesca Clementina Radio, Thiloka Ratnaike (ORCID), Alessandra Renieri, Antonella Riva, Caroline Rooryck, Andreas Roos, Claudia A.L. Ruivenkamp, Andreas Rump, Gijs W.E. Santen, Marco Savarese, Marcello Scala, Katherine Schon, Evelin Schröck, Nika Schuermans, Paolo Scudieri, Martha Spilioti, Verena Steinke-Lange, Pasquale Striano, Yves Sznajer, Marco Tartaglia, Rachel Thompson, Aurelien Trimouille, Bjarne Udd, Paolo Uva, Laura Valle, Lars van der Veken, Roxane van Heurck, Joris van Montfrans, Erika Van Nieuwenhove, Hannah Verdin, David Webb, Brunhilde Wirth, Vicente A. Yépez, Ioannis Zaganas, Federico Zara, and Kristina Zguro.

Acknowledgments

R.H. is supported by a Wellcome Discovery Award (226653/Z/22/Z); the Medical Research Council (UK) (MRC; MR/V009346/1); the Addenbrooke's Charitable Trust, Cambridge University Hospitals (G100142); the Hereditary Neuropathy Foundation; the Evelyn Trust; the Stoneygate Trust; the Lily Foundation; Ataxia UK; Action for AT; the Muscular Dystrophy UK; and the LifeArc Centre to Treat Mitochondrial Diseases (LAC-TreatMito). She is also supported by an MRC strategic award to establish an International Center for Genomic Medicine in Neuromuscular Diseases (ICGNMD) (MR/S005021/1). This research was supported by the NIHR Cambridge Biomedical Research Center (BRC-1215-20014). The views expressed are those of the authors and not necessarily those of the NIHR or the Department of Health and Social Care. T.R. is an academic clinical lecturer supported by Health Education England, East Suffolk and North Essex NHS Foundation Trust, and the University of Cambridge. The Solve-RD project has received funding from the European Union's Horizon 2020 research and innovation programme under grant agreement no. 779257. This study makes use of the RD-Connect GPAP, which received funding originally from the European Union Seventh Framework Programme (FP7/2007-2013) under grant agreement no. 305444 and ongoing funding from EJP-RD (grant nos. H2020 779257 and H2020 825575), Instituto de Salud Carlos III (grant nos. 1217 PT13/0001/0044 and PT17/0009/0019; Instituto Nacional de Bioinformática, INB), and ELIXIR Implementation Studies.

Author contributions

Conceptualization, R.H. and L.M.; data curation, T.R., I.P., and C. O.; formal analysis, T.R. and I.P.; methodology, T.R., I.P., L.M., and R.H.; project administration, A.H. and S.B.; writing – original draft, T.R. and I.P.; writing – review & editing, T.R., I.P., C.O., L. M., and R.H.; supervision, L.M. and R.H.

Declaration of interests

The authors declare no competing interests.

Supplemental information

Supplemental information can be found online at https://doi.org/10.1016/j.ajhg.2025.04.003.

Web resources

ClinicalTrials.gov, https://clinicaltrials.gov

European Genome-Phenome Archive, https://ega-archive.org/datasets/

GenBank, https://www.ncbi.nlm.nih.gov/genbank/

HGNC, https://www.genenames.org

MitoPhen, https://mitophen.org/

OMIM, http://www.omim.org

RD-Connect GPAP, https://platform.rd-connect.eu

Received: November 27, 2024
Accepted: April 7, 2025
Published: April 29, 2025

References

1. Schon, K.R., Ratnaike, T., van den Ameele, J., Horvath, R., and Chinnery, P.F. (2020). Mitochondrial Diseases: A Diagnostic Revolution. Trends Genet. *36*, 702–717. https://doi.org/10.1016/j.tig.2020.06.009.
2. Gorman, G.S., Schaefer, A.M., Ng, Y., Gomez, N., Blakely, E. L., Alston, C.L., Feeney, C., Horvath, R., Yu-Wai-Man, P., Chinnery, P.F., et al. (2015). Prevalence of nuclear and mitochondrial DNA mutations related to adult mitochondrial disease. Ann. Neurol. *77*, 753–759. https://doi.org/10.1002/ana.24362.
3. Laricchia, K.M., Lake, N.J., Watts, N.A., Shand, M., Haessly, A., Gauthier, L., Benjamin, D., Banks, E., Soto, J., Garimella, K., et al. (2022). Mitochondrial DNA variation across 56,434 individuals in gnomAD. Genome Res. *32*, 569–582. https://doi.org/10.1101/gr.276013.121.
4. Hubens, W.H.G., Vallbona-Garcia, A., de Coo, I.F.M., van Tienen, F.H.J., Webers, C.A.B., Smeets, H.J.M., and Gorgels, T.G.M.F. (2022). Blood biomarkers for assessment of mitochondrial dysfunction: An expert review. Mitochondrion *62*, 187–204. https://doi.org/10.1016/j.mito.2021.10.008.

5. Mavraki, E., Labrum, R., Sergeant, K., Alston, C.L., Woodward, C., Smith, C., Knowles, C.V.Y., Patel, Y., Hodsdon, P., Baines, J.P., et al. (2023). Genetic testing for mitochondrial disease: the United Kingdom best practice guidelines. Eur. J. Hum. Genet. *31*, 148–163. https://doi.org/10.1038/s41431-022-01249-w.
6. Ratnaike, T.E., Greene, D., Wei, W., Sanchis-Juan, A., Schon, K.R., van den Ameele, J., Raymond, L., Horvath, R., Turro, E., and Chinnery, P.F. (2021). MitoPhen database: a human phenotype ontology-based approach to identify mitochondrial DNA diseases. Nucleic Acids Res. *49*, 9686–9695. https://doi.org/10.1093/nar/gkab726.
7. Zurek, B., Ellwanger, K., Vissers, L.E.L.M., Schüle, R., Synofzik, M., Töpf, A., de Voer, R.M., Laurie, S., Matalonga, L., Gilissen, C., et al. (2021). Solve-RD: systematic pan-European data sharing and collaborative analysis to solve rare diseases. Eur. J. Hum. Genet. *29*, 1325–1331. https://doi.org/10.1038/s41431-021-00859-0.
8. Matalonga, L., Hernández-Ferrer, C., Piscia, D., Schüle, R., Schüle, R., Töpf, A., Töpf, A., Vissers, L.E.L.M., de Voer, R., et al.; Solve-RD DITF-GENTURIS (2021). Solving patients with rare diseases through programmatic reanalysis of genome-phenome data. Eur. J. Hum. Genet. *29*, 1337–1347. https://doi.org/10.1038/s41431-021-00852-7.
9. Laurie, S., Piscia, D., Matalonga, L., Corvó, A., Fernández-Callejo, M., Garcia-Linares, C., Hernandez-Ferrer, C., Luengo, C., Martínez, I., Papakonstantinou, A., et al. (2022). The RD-Connect Genome-Phenome Analysis Platform: Accelerating diagnosis, research, and gene discovery for rare diseases. Hum. Mutat. *43*, 717–733. https://doi.org/10.1002/humu.24353.
10. Gargano, M.A., Matentzoglu, N., Coleman, B., Addo-Lartey, E.B., Anagnostopoulos, A.V., Anderton, J., Avillach, P., Bagley, A.M., Bakštein, E., Balhoff, J.P., et al. (2024). The Human Phenotype Ontology in 2024: phenotypes around the world. Nucleic Acids Res. *52*. D1333-d1346. https://doi.org/10.1093/nar/gkad1005.
11. Peng, J., Li, Q., and Shang, X. (2017). Investigations on factors influencing HPO-based semantic similarity calculation. J. Biomed. Semant. *8*, 34. https://doi.org/10.1186/s13326-017-0144-y.
12. Hamosh, A., Scott, A.F., Amberger, J.S., Bocchini, C.A., and McKusick, V.A. (2005). Online Mendelian Inheritance in Man (OMIM), a knowledgebase of human genes and genetic disorders. Nucleic Acids Res. *33*, D514–D517. https://doi.org/10.1093/nar/gki033.
13. Griffin, H.R., Pyle, A., Blakely, E.L., Alston, C.L., Duff, J., Hudson, G., Horvath, R., Wilson, I.J., Santibanez-Koref, M., Taylor, R.W., and Chinnery, P.F. (2014). Accurate mitochondrial DNA sequencing using off-target reads provides a single test to identify pathogenic point mutations. Genet. Med. *16*, 962–971. https://doi.org/10.1038/gim.2014.66.
14. Santorsola, M., Calabrese, C., Girolimetti, G., Diroma, M.A., Gasparre, G., and Attimonelli, M. (2016). A multi-parametric workflow for the prioritization of mitochondrial DNA variants of clinical interest. Hum. Genet. *135*, 121–136. https://doi.org/10.1007/s00439-015-1615-9.
15. Benson, D.A., Cavanaugh, M., Clark, K., Karsch-Mizrachi, I., Ostell, J., Pruitt, K.D., and Sayers, E.W. (2018). GenBank. Nucleic Acids Res. *46*, D41–D47. https://doi.org/10.1093/nar/gkx1094.
16. Greene, D., Richardson, S., and Turro, E. (2017). ontologyX: a suite of R packages for working with ontological data. Bioinformatics *33*, 1104–1106. https://doi.org/10.1093/bioinformatics/btw763.
17. Wickham, H. (2022). ggplot2: Elegant Graphics for Data Analysis (Springer-Verlag).
18. Warnes, G.R., Bolker, B., Bonebakker, L., Gentleman, R., Huber, W., Liaw, A., Lumley, T., Maechler, M., Magnusson, A., and Moeller, S. (2009). gplots: Various R programming tools for plotting data. R package version *2*, 1.
19. Yu-Wai-Man, P., Griffiths, P.G., Brown, D.T., Howell, N., Turnbull, D.M., and Chinnery, P.F. (2003). The epidemiology of Leber hereditary optic neuropathy in the North East of England. Am. J. Hum. Genet. *72*, 333–339. https://doi.org/10.1086/346066.
20. Barbarino, J.M., McGregor, T.L., Altman, R.B., and Klein, T.E. (2016). PharmGKB summary: very important pharmacogene information for MT-RNR1. Pharmacogenet. Genom. *26*, 558–567. https://doi.org/10.1097/fpc.0000000000000247.
21. Robin, X., Turck, N., Hainard, A., Tiberti, N., Lisacek, F., Sanchez, J.C., and Müller, M. (2011). pROC: an open-source package for R and S+ to analyze and compare ROC curves. BMC Bioinf. *12*, 77. https://doi.org/10.1186/1471-2105-12-77.
22. Bradley, A.P. (1997). The use of the area under the ROC curve in the evaluation of machine learning algorithms. Pattern Recogn. *30*, 1145–1159. https://doi.org/10.1016/S0031-3203(96)00142-2.
23. Martin, A.R., Williams, E., Foulger, R.E., Leigh, S., Daugherty, L.C., Niblock, O., Leong, I.U.S., Smith, K.R., Gerasimenko, O., Haraldsdottir, E., et al. (2019). PanelApp crowdsources expert knowledge to establish consensus diagnostic gene panels. Nat. Genet. *51*, 1560–1565. https://doi.org/10.1038/s41588-019-0528-2.
24. Martínez-Camblor, P., and Pardo-Fernández, J.C. (2019). The Youden Index in the Generalized Receiver Operating Characteristic Curve Context. Int. J. Biostat. *15*, 20180060. https://doi.org/10.1515/ijb-2018-0060.
25. McCormick, E.M., Lott, M.T., Dulik, M.C., Shen, L., Attimonelli, M., Vitale, O., Karaa, A., Bai, R., Pineda-Alvarez, D.E., Singh, L.N., et al. (2020). Specifications of the ACMG/AMP standards and guidelines for mitochondrial DNA variant interpretation. Hum. Mutat. *41*, 2028–2057. https://doi.org/10.1002/humu.24107.
26. Hajian-Tilaki, K. (2013). Receiver Operating Characteristic (ROC) Curve Analysis for Medical Diagnostic Test Evaluation. Caspian J. Intern. Med. *4*, 627–635.
27. Dunn, O.J. (1961). Multiple Comparisons among Means. J. Am. Stat. Assoc. *56*, 52–64. https://doi.org/10.1080/01621459.1961.10482090.
28. Haynes, W. (2013). Bonferroni Correction. In Encyclopedia of Systems Biology, W. Dubitzky, O. Wolkenhauer, K.-H. Cho, and H. Yokota, eds. (Springer New York), p. 154. https://doi.org/10.1007/978-1-4419-9863-7_1213.
29. Olimpio, C., Paramonov, I., Matalonga, L., Laurie, S., Schon, K., Polavarapu, K., Kirschner, J., Schara-Schmidt, U., Lochmüller, H., Chinnery, P.F., and Horvath, R. (2024). Increased Diagnostic Yield by Reanalysis of Whole Exome Sequencing Data in Mitochondrial Disease. J. Neuromuscul. Dis. *11*, 767–775. https://doi.org/10.3233/jnd-240020.
30. Grady, J.P., Pickett, S.J., Ng, Y.S., Alston, C.L., Blakely, E.L., Hardy, S.A., Feeney, C.L., Bright, A.A., Schaefer, A.M., Gorman, G.S., et al. (2018). mtDNA heteroplasmy level and

copy number indicate disease burden in m.3243A>G mitochondrial disease. EMBO Mol. Med. *10*, e8262. https://doi.org/10.15252/emmm.201708262.

31. Cannon, S.J., Hall, T., Hawkes, G., Colclough, K., Boggan, R. M., Wright, C.F., Pickett, S.J., Hattersley, A.T., Weedon, M.N., and Patel, K.A. (2024). Penetrance and expressivity of mitochondrial variants in a large clinically unselected population. Hum. Mol. Genet. *33*, 465–474. https://doi.org/10.1093/hmg/ddad194.
32. Schon, K.R., Horvath, R., Wei, W., Calabrese, C., Tucci, A., Ibañez, K., Ratnaike, T., Pitceathly, R.D.S., Bugiardini, E., Quinlivan, R., et al. (2021). Use of whole genome sequencing to determine genetic basis of suspected mitochondrial disorders: cohort study. Bmj *375*, e066288. https://doi.org/10.1136/bmj-2021-066288.
33. Stenton, S.L., Laricchia, K., Lake, N.J., Chaluvadi, S., Ganesh, V., DiTroia, S., Osei-Owusu, I., Pais, L., O'Heir, E., Austin-Tse, C., et al. (2024). Mitochondrial DNA variant detection in over 6,500 rare disease families by the systematic analysis of exome and genome sequencing data resolves undiagnosed cases. Preprint at medRxiv. https://doi.org/10.1101/2024.12.22.24319370.
34. Gao, F., Schon, K.R., Vandrovcova, J., Wilson, L., Hanna, M. G., Çavdarlı, B., Çavdarlı, B., Heckmann, J., Chinnery, P.F., and Horvath, R. (2024). Mitochondrial DNA disorders in neuromuscular diseases in diverse populations. Ann. Clin. Transl. Neurol. https://doi.org/10.1002/acn3.52141.
35. Wei, W., Schon, K.R., Elgar, G., Orioli, A., Tanguy, M., Giess, A., Tischkowitz, M., Caulfield, M.J., and Chinnery, P.F. (2022). Nuclear-embedded mitochondrial DNA sequences in 66,083 human genomes. Nature *611*, 105–114. https://doi.org/10.1038/s41586-022-05288-7.
36. Meng, F., He, Z., Tang, X., Zheng, J., Jin, X., Zhu, Y., Ren, X., Zhou, M., Wang, M., Gong, S., et al. (2018). Contribution of the tRNAIle 4317A→G mutation to the phenotypic manifestation of the deafness-associated mitochondrial 12S rRNA 1555A→G mutation. J. Biol. Chem. *293*, 3321–3334. https://doi.org/10.1074/jbc.RA117.000530.
37. Wei, W., Gomez-Duran, A., Hudson, G., and Chinnery, P.F. (2017). Background sequence characteristics influence the occurrence and severity of disease-causing mtDNA mutations. PLoS Genet. *13*, e1007126. https://doi.org/10.1371/journal.pgen.1007126.
38. Davis, R.L., Kumar, K.R., Puttick, C., Liang, C., Ahmad, K.E., Edema-Hildebrand, F., Park, J.S., Minoche, A.E., Gayevskiy, V., Mallawaarachchi, A.C., et al. (2022). Use of Whole-Genome Sequencing for Mitochondrial Disease Diagnosis. Neurology *99*, e730–e742. https://doi.org/10.1212/wnl.0000000000200745.
39. Laurie, S., Steyaert, W., de Boer, E., Polavarapu, K., Schuermans, N., Sommer, A.K., Demidov, G., Ellwanger, K., Paramonov, I., Thomas, C., et al. (2025). Genomic reanalysis of a pan-European rare-disease resource yields new diagnoses. Nat. Med. *31*, 478–489. https://doi.org/10.1038/s41591-024-03420-w.

The American Journal of Human Genetics, Volume *112*

Supplemental information

Mitochondrial DNA disease discovery through evaluation of genotype and phenotype data: The Solve-RD experience

Thiloka Ratnaike, Ida Paramonov, Catarina Olimpio, Alexander Hoischen, Sergi Beltran, Leslie Matalonga, Solve-RD Consortium, and Rita Horváth

Supplemental Information

Supplemental Note: Case Reports

Case summaries for individuals with likely causative variants supported by phenotype similarity score

The following data is summarized in **Table S2. P22** carries *NC_012920.1:m.3302A>G*, and presented with epileptic encephalopathy, epileptic spasm, microcephaly, and global developmental delay. The variant was found at 86% heteroplasmy in blood, with a medium phenotype similarity score (0.41), and a recent publication presented a similar individual with myoclonic epilepsy with ragged red fibers (MERRF, OMIM:545000),[8] therefore the variant has been fed back to the recruiting clinical team.

P23, carrying *NC_012920.1:m.8344A>G*, presented with mitochondrial myopathy, progressive muscle weakness, ptosis, and myopathic electromyography. There were also additional terms including hemifacial atrophy and arrhythmia which are not classically seen with MERRF (OMIM:545000), the commonly associated condition with this variant.[8] The blood heteroplasmy was 71%, with a high phenotype similarity (0.53), so the clinical team were made aware of this likely diagnosis.

P24 with *NC_012920.1:m.8993T>G* had an infantile presentation of epilepsy, cerebellar atrophy, increased serum lactate and arachnoid cyst. There was a high blood heteroplasmy of 94%, with medium phenotype similarity (0.44) likely due to the additional phenotype term of 'arachnoid cyst', which is probably not related to mitochondrial disease. Given the likelihood of maternally inherited Leigh syndrome[9] in this case (OMIM:500017), it was urgently fed back to the clinical team.

Single system conditions: **P25-27** were found to have near-homoplasmic variants known to cause LHON[10] (OMIM:535000), with high phenotype similarity in each case (1.0): *NC_012920.1:m.11778G>A, NC_012920.1:m.14484T>C, NC_012920.1:m.3460G>A.* **P28-37** were found to have HPO terms related to sensorineural hearing impairment leading to high phenotype similarity (0.57-0.58). The variant *NC_012920.1:m.1555A>G* was found at 93%-100% blood heteroplasmy levels. One individual was noted to have a phenotype extending beyond hearing impairment with microcephaly and global developmental delay, therefore the *NC_012920.1:m.1555A>G* is only partially explaining this person's phenotype. The variants were fed back via the DITF, and the data is available in **Table S2**.

Variants not in MitoPhen

There were 29 individuals with variants not found in MitoPhen (P19-21 and P38-63 in **Table S2**). 6/29 (21%) had high PSS (>0.5) although the variants were classified as VUS, 2/6 (33%) were diagnostic (P19 and P20, **Table S2**). The variants identified in individuals 19 and 20 in our pipeline, classified as VUS by our team, had also been tagged as causative in the GPAP in parallel. Individual 19 was diagnosed with the *NC_012920.1:m.5698G>A* variant with a blood heteroplasmy of 49%, and a high PSS (0.64) due to a phenotype in keeping with a mitochondrial myopathy including terms: ragged-red muscle fibers, ptosis, external ophthalmoplegia and proximal muscle weakness. A previous study reported a person with a similar presentation, with the single muscle fiber study confirming segregation of the variant to fibers with mitochondrial dysfunction.[11] Therefore, the individual in our study is the second to be reported with the same diagnosis.[12] P20 was diagnosed with the *NC_012920.1:m.9032T>C* variant at 84% blood heteroplasmy, high PSS (0.51) with phenotypic terms indicating a multisystem condition: muscular hypotonia, motor delay,

constipation, feeding difficulties in infancy, and bruxism. Previous publications have indicated that this variant is associated with neurogenic muscle weakness, ataxia and retinitis pigmentosa[13] or a neurodevelopmental disorder with a broader spectrum,[14,15] at high blood heteroplasmy levels.

P38 carried the *NC_012920.1:m.16023G>A* variant at 7.6% blood heteroplasmy, with a high PSS (0.92) due to a range of phenotypes including hearing impairment, pes cavus, cardiac anomalies, mild global developmental delay, cleft palate, and hypospadias. This variant was classified as a VUS and we have reported this finding back to the referring team to gain further information about segregation of the variant. Previous reports of this variant suggest differing phenotypic spectrums.[16,17] P39, who presented with hypotonia and seizures, was found to have the *NC_012920.1:m.3249G>A* variant, at a blood heteroplasmy of 4.2%. This variant was also classified as a VUS.

Of the 2 remaining individuals with variants associated with high PSS, one variant was classified as likely benign (P40 with *NC_012920.1:m.14696A>G*, PSS was 0.62, heteroplasmy 8%), and P41 had an alternative diagnosis (nuclear gene variant).

There were 19/29 (66%) individuals with a medium PSS. Of note, P21 was diagnosed with the homoplasmic *NC_012920.1:m.9478T>C* variant by the local laboratory associated with a medium PSS (0.33). Although this variant has been reported previously to segregate with Leigh syndrome[18] and APOGEE 2[5] predicts this variant as deleterious, there were no additional lines of evidence to support pathogenicity,[19] so it remains classified a VUS. The *NC_012920.1:m.15990C>T* variant identified in P42 with a PSS of 0.48, was the only variant with a likely pathogenic classification in MITOMAP and by the ClinGen Mitochondrial Disease Nuclear and Mitochondrial Variant Curation Expert Panel classification on ClinVar.[7] However, this variant was at 4% heteroplasmy and the person had an alternative nuclear

genetic diagnosis noted on RD-Connect GPAP. The remaining 4/29 (14%) individuals had low PSS, and the variants were all classified as VUS, so they remain undiagnosed (**Table S2**).

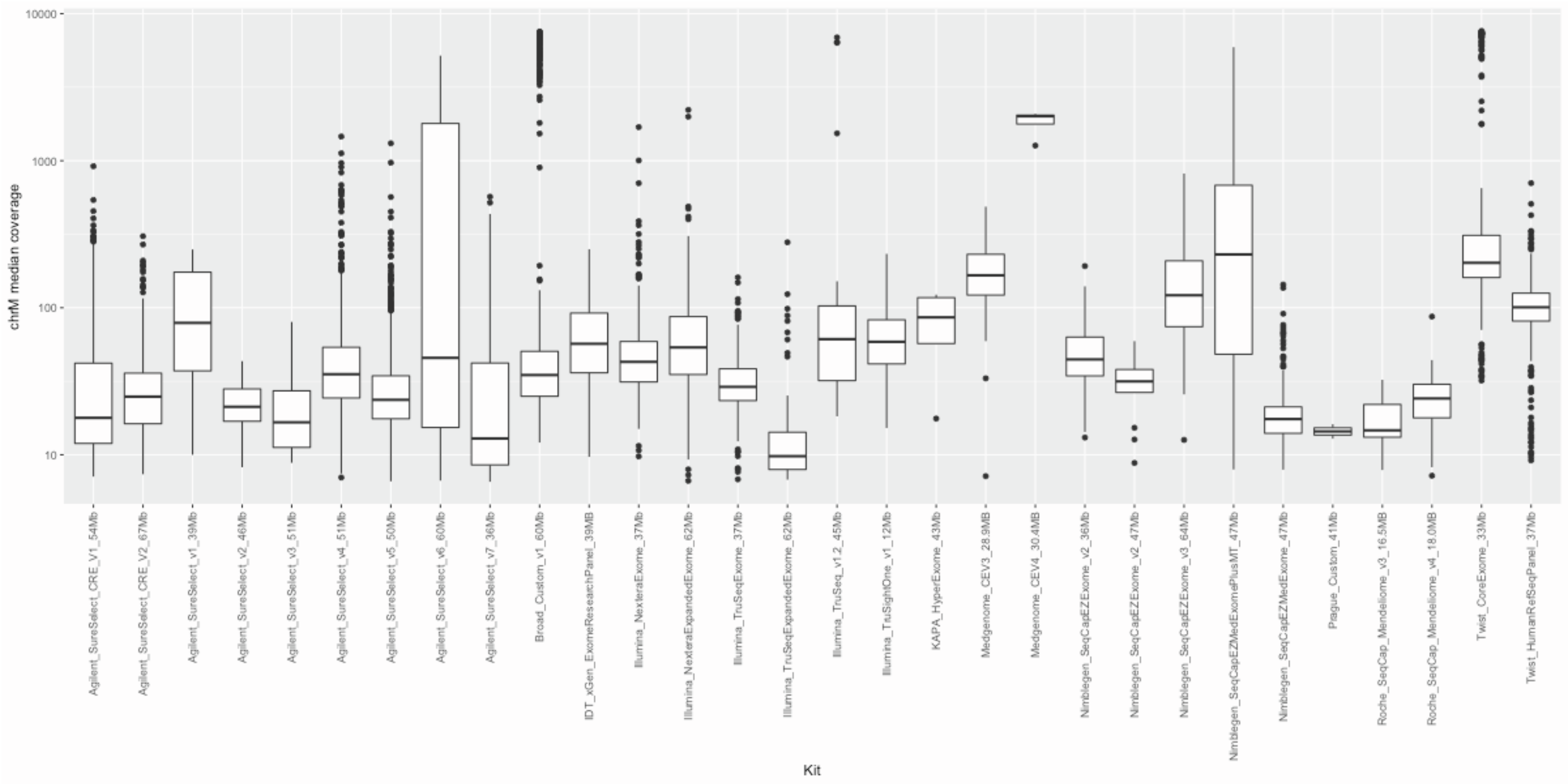

Figure S1: MtDNA coverage across different Exome sequencing (ES) kits after QC filtering.

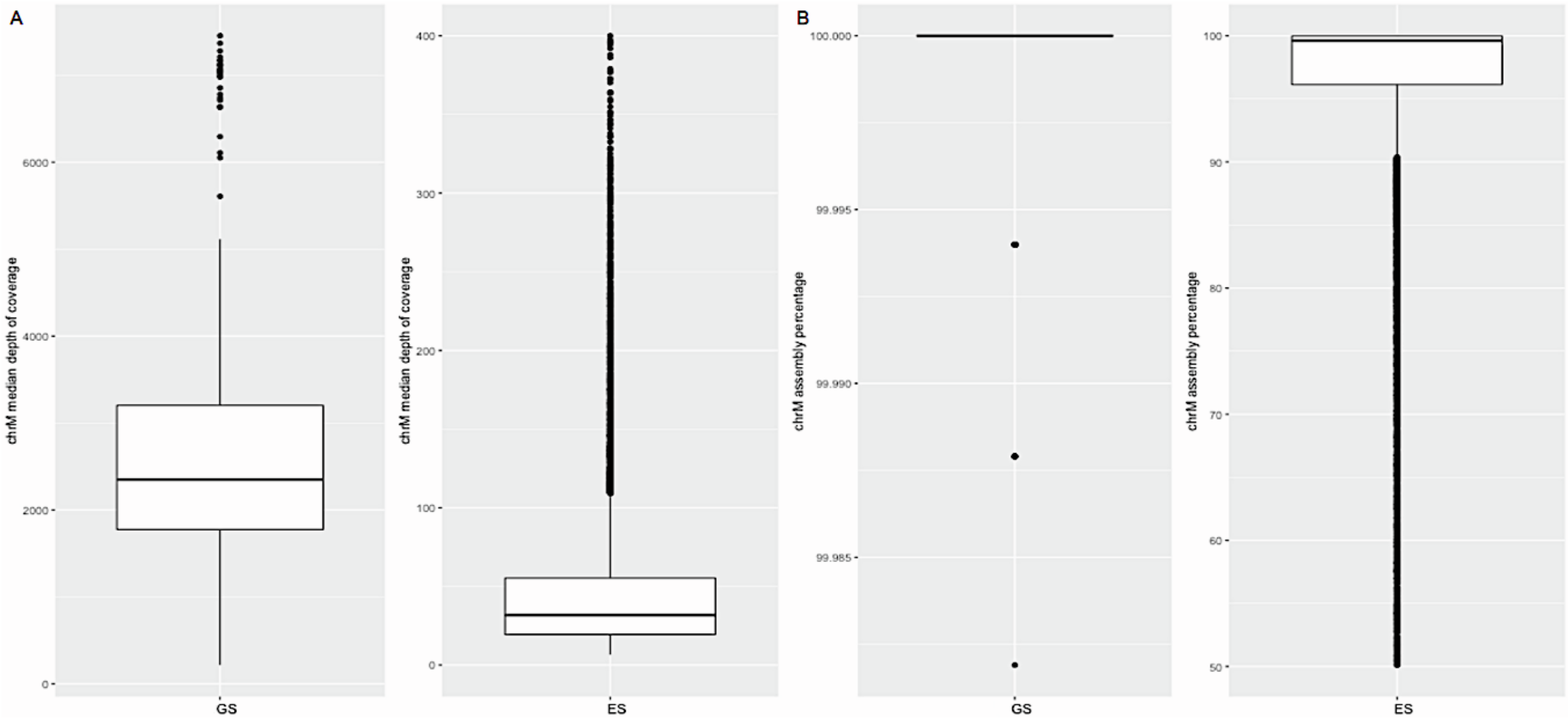

Figure S2: MtDNA coverage and assembly percentage in exome sequencing (ES) and genome sequencing (GS) after QC filtering. A. Median mtDNA coverage in GS (n=523) and ES (n=9,634). B. Percentage of mtDNA assembled in GS (n=523) and ES (n=9,634).

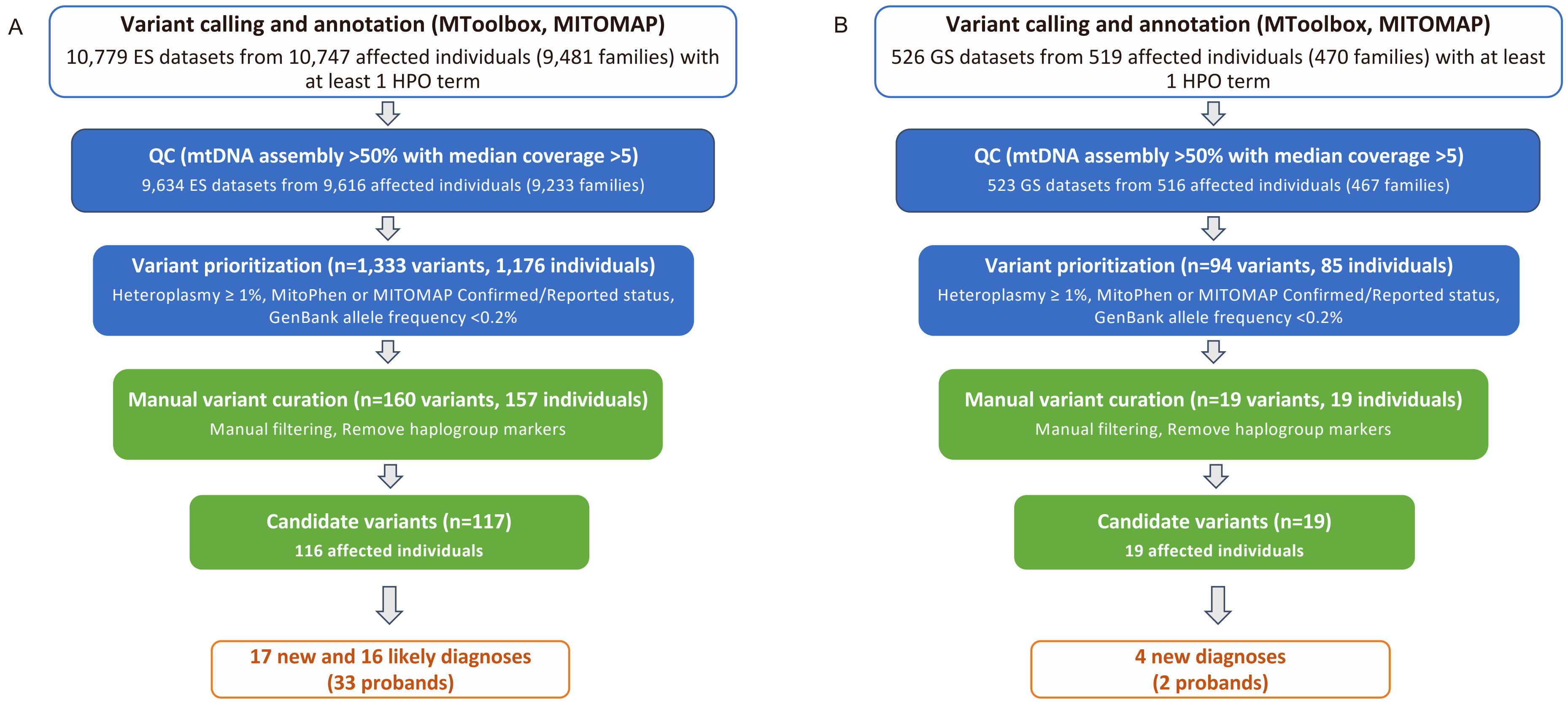

Figure S3: MtDNA variant analysis workflow in exome sequencing (ES) and genome sequencing (GS) datasets. The numbers listed are the number of distinct individual-variant combinations found. Blue and green boxes highlight automated and manual prioritization approaches respectively, with orange highlighting diagnostic/likely causative mtDNA variants. A. Prioritization workflow in 10,779 ES datasets. B. Prioritization workflow in 526 GS datasets.

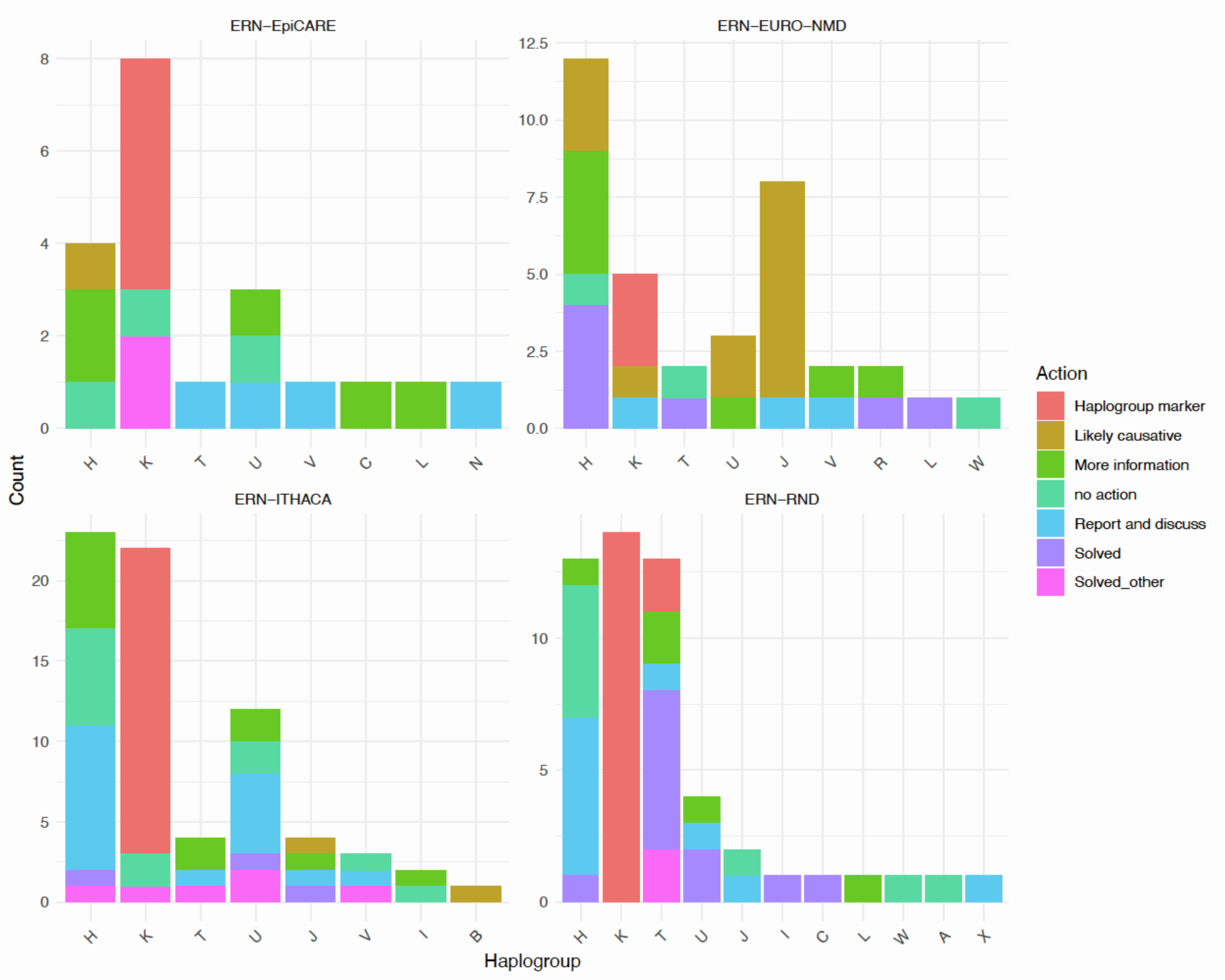

Figure S4: MtDNA haplogroup analysis by ERN. Macro-haplogroup data was used and bars are colored by the final action taken per individual (n=176). The most common haplogroups in the dataset were H, K, J and T. The variant *m.4295A>G* was associated with haplogroup K, and *m.4317A>G* was associated with haplogroup T.

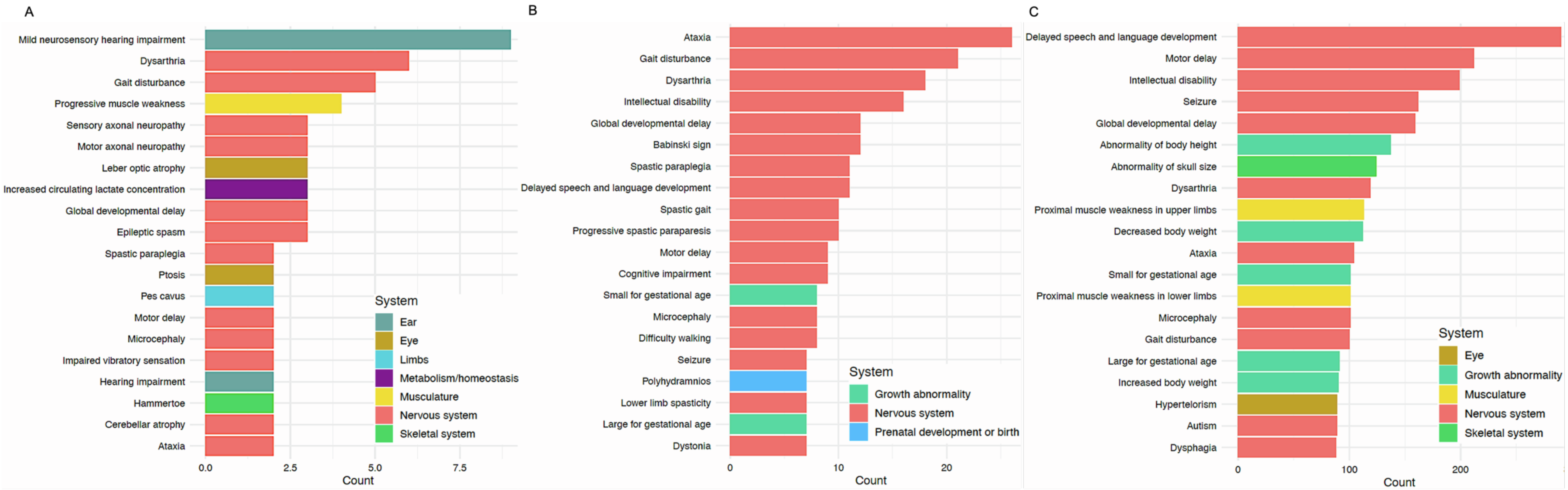

Figure S5: Top 20 frequent HPOs, based on non-redundant terms per diagnosed individual, by genetic diagnosis. A: MtDNA diagnoses (n=37), **B**: Nuclear-mitochondrial gene diagnoses (n=122), **C**: Nuclear-other gene diagnoses (n=1,338).

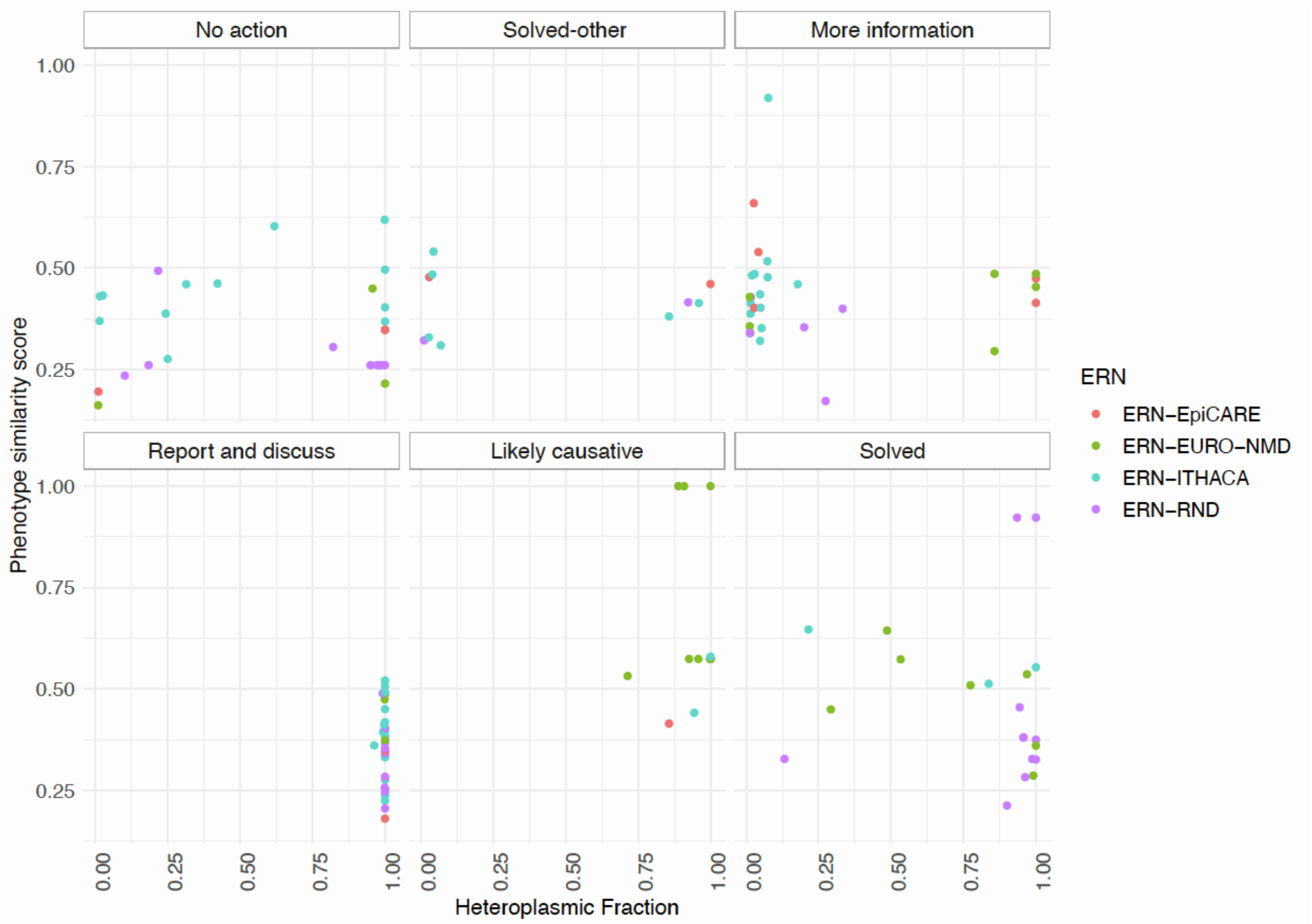

Figure S6: Phenotype similarity score, heteroplasmy level per mtDNA variant, and action taken per sample. The points are colored by ERN that each individual was recruited through.

HGVS nomenclature	Gene, HGNC ID	HF	PSS	ChrM assembled range	Mean chrM coverage range	Number of individuals	ACMG classification (source)
NC_012920.1:m.11778G>A	MT-ND4, HGNC:7459	0.333	1	99.825 - 99.825	42.925 - 42.925	1	Pathogenic (ClinGen)
NC_012920.1:m.11778G>A	MT-ND4, HGNC:7459	0.821	1	99.8914 - 99.8914	24.51 - 24.51	1	Pathogenic (ClinGen)
NC_012920.1:m.11778G>A	MT-ND4, HGNC:7459	0.969	1	99.8733 - 99.8733	40.702 - 40.702	1	Pathogenic (ClinGen)
NC_012920.1:m.11778G>A	MT-ND4, HGNC:7459	1	1	62.46 - 99.994	7.279 - 56.352	7	Pathogenic (ClinGen)
NC_012920.1:m.14484T>C	MT-ND6, HGNC:7462	0.617	1	99.825 - 99.825	42.925 - 42.925	1	Pathogenic (ClinGen)
NC_012920.1:m.14484T>C	MT-ND6, HGNC:7462	1	1	99.6258 - 99.6258	47.353 - 47.353	1	Pathogenic (ClinGen)
NC_012920.1:m.14495A>G	MT-ND6, HGNC:7462	0.935	1	99.9819 - 99.9819	50.729 - 50.729	1	Likely pathogenic (ClinGen)
NC_012920.1:m.14495A>G	MT-ND6, HGNC:7462	0.977	1	99.5896 - 99.5896	33.457 - 33.457	1	Likely pathogenic (ClinGen)
NC_012920.1:m.14674T>C	MT-TE, HGNC:7479	0.98	0.34	99.7707 - 99.7707	68.967 - 68.967	1	Likely pathogenic (ClinGen)
NC_012920.1:m.14674T>C	MT-TE, HGNC:7479	1	0.34	65.3751 - 99.994	13.101 - 165.35	8	Likely pathogenic (ClinGen)
NC_012920.1:m.14674T>C	MT-TE, HGNC:7479	1	0.49	90.1141 - 99.994	17.358 - 155.755	11	Likely pathogenic (ClinGen)
NC_012920.1:m.3460G>A	MT-ND1, HGNC:7455	0.969	1	99.837 - 99.837	56.785 - 56.785	1	Pathogenic (ClinGen)
NC_012920.1:m.3460G>A	MT-ND1, HGNC:7455	1	1	94.1879 - 99.8914	11.75 - 58.473	8	Pathogenic (ClinGen)

Table S1: MtDNA variant benchmarking in 42 individuals with previously confirmed genetic diagnoses (43 confirmed variants – one individual was diagnosed with variants *NC_012920.1:m.11778G>A* and *NC_012920.1:m.14484T>C*). Table is grouped by mtDNA variant, number of individuals are shown per group. HF: heteroplasmic fraction; PSS: phenotype similarity scores; ChrM: Chromosome M or mitochondrial genome.

Supplemental Methods

Mitochondrial DNA manual curation

For mitochondrial DNA (mtDNA) variants not found in MitoPhen, we applied ACMG criteria and tRNA pathogenicity criteria where applicable.[1,2] For pathogenicity assessments, MITOMAP[3], Mitomaster[4] and the Franklin (http://franklin.genoox.com) tool were used to search for publications related to each variant as well as allele frequencies. APOGEE 2[5] and MitoTip[6] were used to determine pathogenicity predictions for variants. Additionally, ClinVar[7], Pubmed and Google searches were conducted per variant of interest to identify any recent publications of individual-level information.

Supplemental Results

MtDNA analysis in the unsolved cohort

From the 11,305 datasets analyzed in the unsolved cohort, 10,157 (90%) have met our quality inclusion criteria with variable coverage across different ES kits (**Figure S1**). In fact, in 523 out of 526 GS initially included in this study, mtDNA was assembled at 100% and median coverage was 2,347 across all (**Figure S2, Figure S3**). From the 10,779 exomes, 9,634 (89%) datasets were considered for further analysis (**Figure S3**). As shown in Figure S2, median mtDNA assembly was 99.6% and median mtDNA coverage was 34 across ES datasets. A small fraction of these exomes (219/9,634, 2.3%) was sequenced using a kit that enables target enrichment of the mitochondrial genome (Nimblegen_SeqCapEZMedExomePlusMT_47Mb). A median coverage across this group was 230, which is higher than in exomes sequenced by other kits (**Figure S1**). Only 6.5% of ES data overall (518/9,634) showed median coverage below 10.

179 variants were identified in 176 affected individuals using the automated prioritization approach initially, prior to exclusion of haplogroup markers. 160 of the prioritized variants were identified using ES and the other 19 were detected from GS (**Figure S3**). The heteroplasmy varied from 1 to 100% both in ES and GS datasets, while median coverage ranged from 8 to 7,369 in ES, and from 1,125 to 6,780 in GS.

Haplogroup analyses

We performed mtDNA haplogroup analysis to identify any haplogroup markers in the dataset at the first step of manual variant curation (**Figure 1**). There were 179 variants considered in 176 individuals: 43 were mtDNA haplogroup markers, with *NC_012920.1:m.4295A>G* being the most frequent, found in 36 individuals associated with haplogroup K, recruited across the four ERNs (**Figure S4**). Therefore, these variants were discarded from further analysis, resulting in 136 rare mtDNA variants in 135 individuals, for manual curation including phenotype evaluation. 5/135 individuals (4%) belonged to Asian (haplogroup C) or African mtDNA lineages (haplogroup L), as denoted in MITOMAP[3], while the rest belonged to mtDNA lineages associated with European ancestry.

Phenotype similarity analysis

When considering all diagnosed individuals within Solve-RD, the frequently documented HPO terms showed that individuals with a mtDNA disease diagnosis tended to have multisystem involvement (**Figure S5A**), compared to those with other nuclear genetic diseases including nuclear-mitochondrial gene diagnoses (**Figure S5B-C**) where phenotypes included more neurodevelopmental presentations such as intellectual disability. Terms related to sensorineural hearing impairment and neuromuscular presentations such as

‘dysarthria’, ‘gait ataxia’ and ‘progressive muscle weakness’, were most frequent in the mtDNA disease cohort. Individuals with a nuclear-mitochondrial genetic diagnosis had a predominance of HPO terms related to nervous system and neurodevelopmental abnormalities, whereas there were more growth abnormality terms seen in the ‘nuclear-other’ group compared with the other two groups. These differences impact the phenotype similarity score (PSS) calculations and enabled the detection of suspected mtDNA diseases within the heterogeneous Solve-RD cohort.

PSS was evaluated alongside mtDNA variant heteroplasmy data in the 136 variants from 135 affected, unsolved individuals that underwent manual curation. 26/136 variants were classified ‘no further action’: 9/26 were variants of uncertain significance (VUS), and reported back to the DITF, but either did not show segregation of variant within the family, or the PSS was low and the phenotype did not match any previous reports; 8/26 were *NC_012920.1:m.1555A>G* with no associated hearing impairment phenotypes and not found in homoplasmy; 9/26 had medium-low PSS associated with variants which were not reported back to the DITF due to conflicting evidence of pathogenicity (*NC_012920.1:m.4317A>G*, *NC_012920.1:m.4295A>G*) or phenotype not matching previous reports (*NC_012920.1:m.15498G>A*).

Of the 37 individuals who had confirmed mtDNA disease diagnoses (n=21) or likely diagnoses (n=16), 34 (92%) had PSS which were >0.3, and all individuals had mtDNA variant heteroplasmy levels >11%. (**Table S2**, **Figure S6**).

MitoPhen update

NC_012920.1:m.4317 will be removed from MitoPhen pathogenic variant list as there is no new supporting evidence, and it is classified as likely benign/benign. The variant classified as likely pathogenic (*NC_012920.1:m.15990C>T*) will be added to the database.

References

1. McCormick EM, Lott MT, Dulik MC, et al. Specifications of the ACMG/AMP standards and guidelines for mitochondrial DNA variant interpretation. *Human Mutation*. 2020;41(12):2028-2057. doi:https://doi.org/10.1002/humu.24107

2. Yarham JW, Al-Dosary M, Blakely EL, et al. A comparative analysis approach to determining the pathogenicity of mitochondrial tRNA mutations. *Hum Mutat*. Nov 2011;32(11):1319-25. doi:10.1002/humu.21575

3. MITOMAP. MITOMAP: A Human Mitochondrial Genome Database. Accessed 15 May, 2019. http://www.mitomap.org

4. Brandon MC, Ruiz-Pesini E, Mishmar D, et al. MITOMASTER: a bioinformatics tool for the analysis of mitochondrial DNA sequences. *Human Mutation*. 2009/01/01 2009;30(1):1-6. doi:https://doi.org/10.1002/humu.20801

5. Bianco SD, Parca L, Petrizzelli F, et al. APOGEE 2: multi-layer machine-learning model for the interpretable prediction of mitochondrial missense variants. *Nat Commun*. 2023/08/19 2023;14(1):5058. doi:10.1038/s41467-023-40797-7

6. Sonney S, Leipzig J, Lott MT, et al. Predicting the pathogenicity of novel variants in mitochondrial tRNA with MitoTIP. *PLOS Computational Biology*. 2017;13(12):e1005867. doi:10.1371/journal.pcbi.1005867

7. Landrum MJ, Lee JM, Benson M, et al. ClinVar: public archive of interpretations of clinically relevant variants. *Nucleic Acids Res*. Jan 4 2016;44(D1):D862-8. doi:10.1093/nar/gkv1222

8. Huang G, Wang Y, Yao D. Myoclonic epilepsy with ragged red fibers syndrome associated with mitochondrial 3302A>G mutation in the MT-TL1 gene: A case report. *Exp Ther Med*. Feb 2023;25(2):87. doi:10.3892/etm.2023.11786

9. Santorelli FM, Shanske S, Macaya A, DeVivo DC, DiMauro S. The mutation at nt 8993 of mitochondrial DNA is a common cause of Leigh's syndrome. *Ann Neurol*. Dec 1993;34(6):827-34. doi:10.1002/ana.410340612

10. Yu-Wai-Man P, Griffiths PG, Brown DT, Howell N, Turnbull DM, Chinnery PF. The epidemiology of Leber hereditary optic neuropathy in the North East of England. *Am J Hum Genet*. Feb 2003;72(2):333-9. doi:10.1086/346066

11. Spinazzola A, Carrara F, Mora M, Zeviani M. Mitochondrial myopathy and ophthalmoplegia in a sporadic patient with the 5698G-->A mitochondrial DNA mutation. *Neuromuscul Disord*. Dec 2004;14(12):815-7. doi:10.1016/j.nmd.2004.09.002

12. Olimpio C, Paramonov I, Matalonga L, et al. Increased Diagnostic Yield by Reanalysis of Whole Exome Sequencing Data in Mitochondrial Disease. *J Neuromuscul Dis*. May 13 2024;doi:10.3233/jnd-240020

13. López-Gallardo E, Emperador S, Solano A, et al. Expanding the clinical phenotypes of MT-ATP6 mutations. *Human molecular genetics*. 2014;23(23):6191-6200. doi:10.1093/hmg/ddu339

14. Turro E, Astle WJ, Megy K, et al. Whole-genome sequencing of patients with rare diseases in a national health system. *Nature*. 2020/07/01 2020;583(7814):96-102. doi:10.1038/s41586-020-2434-2

15. Knight KM, Shelkowitz E, Larson AA, et al. The mitochondrial DNA variant m.9032T > C in MT-ATP6 encoding p.(Leu169Pro) causes a complex mitochondrial neurological syndrome. *Mitochondrion*. Nov 2020;55:8-13. doi:10.1016/j.mito.2020.08.009

16. Blakely EL, Yarham JW, Alston CL, et al. Pathogenic mitochondrial tRNA point mutations: nine novel mutations affirm their importance as a cause of mitochondrial disease. *Hum Mutat*. Sep 2013;34(9):1260-8. doi:10.1002/humu.22358

17. Wang J, Balciuniene J, Diaz-Miranda MA, et al. Advanced approach for comprehensive mtDNA genome testing in mitochondrial disease. *Mol Genet Metab*. Jan 2022;135(1):93-101. doi:10.1016/j.ymgme.2021.12.006

18. Mkaouar-Rebai E, Ellouze E, Chamkha I, Kammoun F, Triki C, Fakhfakh F. Molecular-clinical correlation in a family with a novel heteroplasmic Leigh syndrome missense mutation in the mitochondrial cytochrome c oxidase III gene. *J Child Neurol*. Jan 2011;26(1):12-20. doi:10.1177/0883073810371227

19. McCormick EM, Zolkipli-Cunningham Z, Falk MJ. Mitochondrial disease genetics update: recent insights into the molecular diagnosis and expanding phenotype of primary mitochondrial disease. *Curr Opin Pediatr*. 2018;30(6):714-724. doi:10.1097/MOP.0000000000000686
